# GFAPδ/GFAPα ratio directs astrocytoma gene expression towards a more malignant profile

**DOI:** 10.18632/oncotarget.21540

**Published:** 2017-10-04

**Authors:** Oscar M.J.A. Stassen, Emma J. van Bodegraven, Fabrizio Giuliani, Martina Moeton, Regina Kanski, Jacqueline A. Sluijs, Miriam E. van Strien, Willem Kamphuis, Pierre A.J. Robe, Elly M. Hol

**Affiliations:** ^1^ Netherlands Institute for Neuroscience, an Institute of The Royal Netherlands Academy of Arts and Sciences, 1105 BA Amsterdam, The Netherlands; ^2^ Soft Tissue Engineering and Mechanobiology, Eindhoven University of Technology, 5612 AZ Eindhoven, The Netherlands; ^3^ Department of Translational Neuroscience, Brain Center Rudolf Magnus, University Medical Center Utrecht, 3584 CG Utrecht, The Netherlands; ^4^ Department of Neurology and Neurosurgery, Brain Center Rudolf Magnus, University Medical Center Utrecht, 3584 CG Utrecht, The Netherlands; ^5^ Swammerdam Institute for Life Sciences, Center for Neuroscience, University of Amsterdam, 1098 XH Amsterdam, The Netherlands

**Keywords:** GFAP-isoforms, astrocytoma, transcriptomics, glioma, intermediate filaments

## Abstract

Astrocytomas are the most common malignant brain tumours and are to date incurable. It is unclear how astrocytomas progress into higher malignant grades. The intermediate filament cytoskeleton is emerging as an important regulator of malignancy in several tumours. The majority of the astrocytomas express the intermediate filament protein Glial Fibrillary Acidic Protein (GFAP). Several *GFAP* splice variants have been identified and the main variants expressed in human astrocytoma are the *GFAP*α and *GFAP*δ isoforms. Here we show a significant downregulation of *GFAP*α in grade IV astrocytoma compared to grade II and III, resulting in an increased GFAPδ/*α* ratio. Mimicking this increase in *GFAPδ/α* ratio in astrocytoma cell lines and comparing the subsequent transcriptomic changes with the changes in the patient tumours, we have identified a set of *GFAPδ/α* ratio-regulated high-malignant and low-malignant genes. These genes are involved in cell proliferation and protein phosphorylation, and their expression correlated with patient survival. We additionally show that changing the ratio of *GFAPδ/α*, by targeting *GFAP* expression, affected expression of high-malignant genes. Our data imply that regulating *GFAP* expression and splicing are novel therapeutic targets that need to be considered as a treatment for astrocytoma.

## INTRODUCTION

Astrocytomas are the most common malignant brain tumours, with an incidence of 5.9 per 100 000 in the Netherlands [1]. Astrocytomas develop from astrocytes, adult neural stem cells, or glia progenitors [2]. These tumours are classified into different grades of malignancy based on histological assessment. Grade I (pilocytic astrocytoma) are slowly growing, localized tumours that only very rarely become anaplastic. Grade II (diffuse astrocytoma) are slowly growing tumours that tend to invade the brain diffusely, and sooner or later progress into the anaplastic grade III, or develop hyperplastic neovessels and areas of necrosis and become grade IV (glioblastoma, GBM) tumours [3]. Glioblastomas can also arise *de novo* [4] and are by far the most common and most malignant variant of astrocytoma. Resection surgery followed by additional radio- and chemotherapy increases survival, but disease recurrence is inevitable. The current median survival time after diagnosis is between 4 and 20 months, depending on the age, the clinical condition of the patient, and the treatment [1]. One of the key factors in the poor response to treatment is the heterogeneity of the cells within a single tumour, with different signalling pathways active at the same time. This makes it extremely challenging to target the entire tumour with a single-pathway drug [5]. Therefore, a better knowledge of the molecular mechanisms of tumour cells interacting with their cellular and proteinaceous environment is highly valuable to improve treatment strategies [6] and patient prognosis [7, 8].

The intermediate filament (IF) protein family is a large family of cytoskeletal proteins that is central in the integration of cellular structure and cell signalling [9]. IF proteins are involved in cellular differentiation processes [10], and are involved in the tumour biology of various malignancies. For instance, keratin 14 is essential for the metastasizing invasive front of breast cancer [11], keratin 17 is an important transforming protein in Ewing Sarcoma [12], and vimentin is a marker of the epithelial to mesenchymal transition and induces the characteristic cellular changes of this transition [13, 14]. Vimentin regulates lung cancer cell adhesion through focal adhesions and activates Slug, a transcription factor involved in epithelial mesenchymal transition in breast carcinoma [15, 16]. Thus, in several tumours a change in the composition of the IF-network induces the progression towards a more invasive tumour. As IF proteins have a broad involvement in cellular functioning, regulate several signalling pathways, and are cell- and tissue-specific, they are an interesting potential therapeutic target, as modulating the IF-network can control down stream targets that are involved in tumour malignancy [15].

A loss of GFAP - the typical astrocyte IF protein - in higher grade astrocytomas was described more than 40 years ago [17], and astrocytoma type IV has been characterized into subtypes on the basis of IF expression [18]. The *GFAP* locus encodes for multiple *GFAP*- isoforms, and at least 10 different splice variants are now known to be expressed in the human brain [19, 20]. There are several indications that a change in the stoichiometry of these isoforms correlates with astrocytoma grade. Staining with two different antibodies for either all GFAP-isoforms or for GFAPδ shows a negative correlation of astrocytoma grade with total GFAP but a positive correlation with GFAPδ immunoreactivity [21–23]. GFAPα and GFAPδ are the most abundantly expressed isoforms in the central nervous system and differ only in their C-terminal 41 amino acids, a signalling hotspot in IF proteins due to the high content of phosphorylatable residues [24]. Both isoforms are expressed in several astrocytoma cell lines [25–27]. *GFAPα* encodes the canonical GFAP-isoform and is expressed in astrocytes and highly upregulated in reactive gliosis, whereas GFAPδ is enriched in neural stem cells and subpial astrocytes in the human brain [20, 28, 29]. The two isoforms differ in their 3′UTR and *GFAPδ* contains the alternative exon 7a, which is part of intron 7 and is not present in *GFAPα* [29, 30].

Since various members of the IF protein family are described to be involved in cell-extracellular matrix (ECM) interactions as well as in signalling and differentiation processes involved in tumour malignancy [11, 31, 32], we investigated the expression of *GFAP-* isoforms in astrocytoma. We first analysed *GFAP*-isoform expression in a large RNA sequencing dataset from TCGA and observed different levels of *GFAPα* expression in astrocytomas of different grade as well as a different *GFAPδ/α* ratio. Subsequently, modulation of GFAPα and the GFAPδ/α ratio in an astrocytoma cell model resulted in transcriptional changes, which we related to the observations in patients, and resulted in the identification of a set of high-malignant and low-malignant genes that are regulated by GFAP.

## RESULTS

### *GFAP*-isoform expression differs between low and high grade astrocytoma

**Figure 1:**
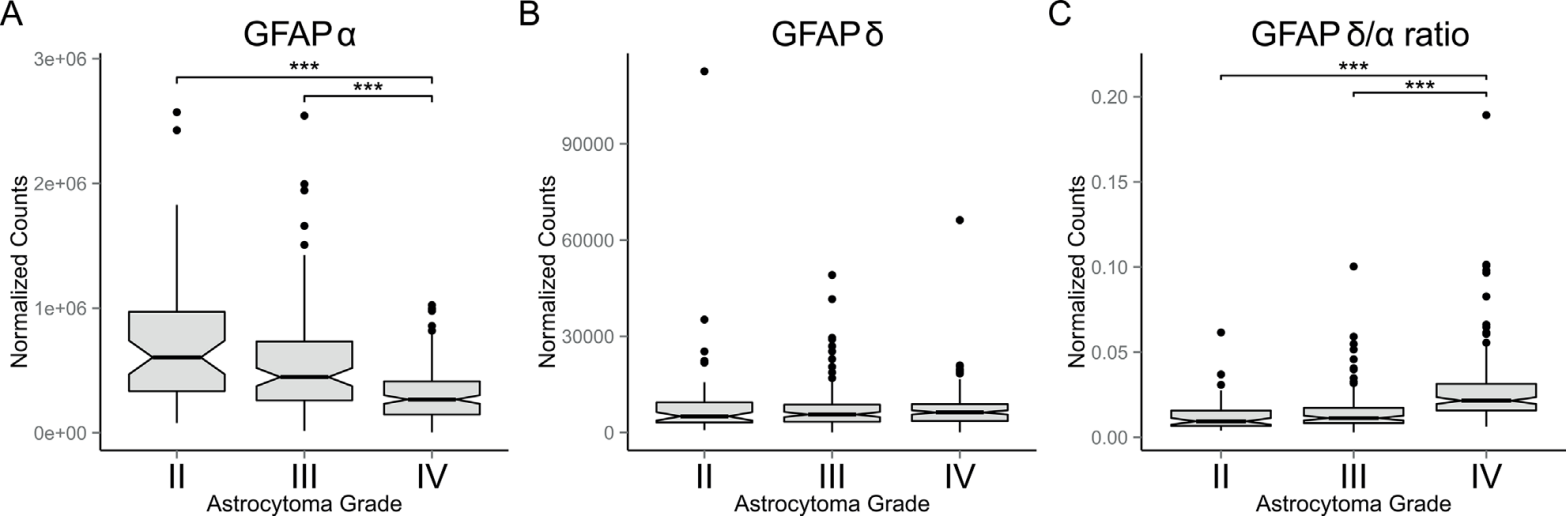
*GFAP*-isoform expression in astrocytoma Box plots showing: **(A)** *GFAPα*, **(B)** *GFAPδ*, and **(C)** *GFAPδ/α* levels in grade II (n=55), III (n=105) and IV (n=150) astrocytomas (*whiskers:* ±1.5 × IQR; *notch:* 95% CI; ^***^: FDR < 0.001). Expression levels of *GFAP*-isoforms were obtained from RNAseq level 3 released normalized isoform expression data of the TCGA database. The *GFAPδ/α* ratio was calculated for each patient. A significant decrease in *GFAPα* expression in grade IV astrocytoma compared to grade II and III (A) and no difference in *GFAPδ* expression between astrocytoma of different grade (B) resulted in a relative increase in GFAPδ expression compared to GFAPα (C), which we define as the *GFAPδ/α* ratio.

To get insight into the biological processes that determine the malignancy of astrocytoma and to investigate the potential role of GFAP-isoforms in regulating these processes, we performed a differential gene expression analysis on RNAseq data of low- and high-grade astrocytoma obtained from TCGA. The final cohort used in our analysis included 150 grade IV, 105 grade III, and 55 grade II astrocytoma. Normalized gene expression data was used to analyse differential gene expression between low- (II and III) and high-grade (IV) astrocytoma. *GFAP* expression was significantly decreased in grade IV compared to both grade II (46%, FDR= 1.58E-10) and grade III (58%, FDR= 3.92E-7) astrocytoma. Interestingly, the absolute difference in normalized gene expression of all genes analysed between low- and high-grade astrocytoma was the largest for *GFAP*. Grade II versus IV showed an absolute difference in *GFAP* expression of 3.57E5 normalized counts and grade III versus IV of 2.20E5 normalized counts. In order to determine the expression levels of the *GFAP*-isoforms, TCGA derived RNA sequencing data consisting of normalized isoform expression data was used. The only known *GFAP*-isoforms that were annotated in this dataset were *GFAPα* and *GFAPδ*. Interestingly, while canonical *GFAPα* expression is indeed significantly decreased in grade IV astrocytoma compared to grade II (45%, FDR = 1.20E-4; Figure 1A) and to grade III (55%, FDR = 8.4E-4; Figure 1A), the expression of the alternative splice variant *GFAPδ* is not different between astrocytoma grades (Figure 1B). This results in a relative increase in *GFAPδ* compared to *GFAPα*, which we report as the *GFAPδ/α* ratio. Importantly, the *GFAPδ/α* ratio was significantly increased in grade IV astrocytoma compared to both grade II (220%, FDR = 5.87E-08; Figure 1C) and grade III (177%, FDR = 4.16E-08; Figure 1C).

### Extracellular matrix genes are overrepresented in transcriptome changes due to GFAP modulation in astrocytoma cell lines *in vitro*

**Figure 2:**
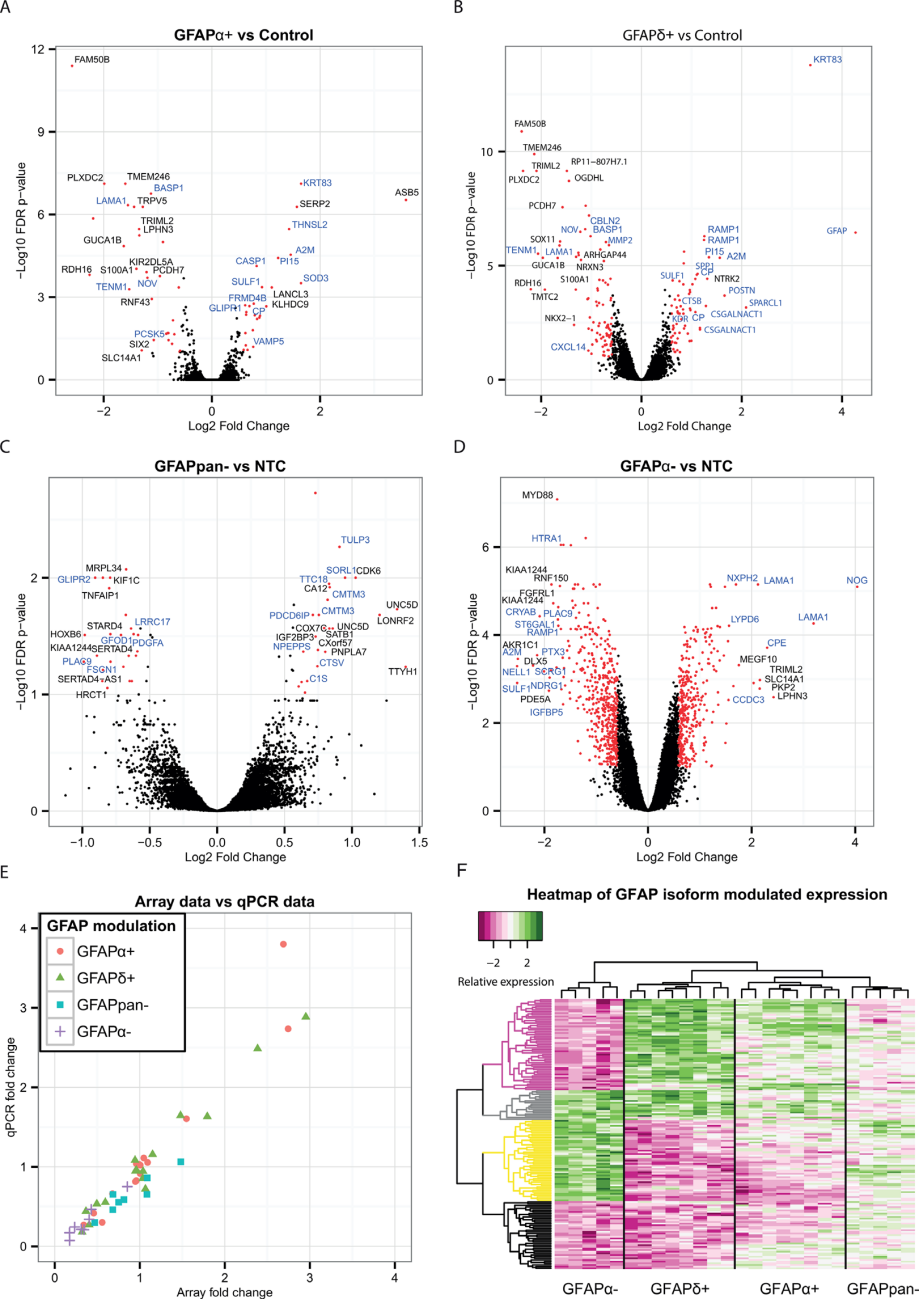
Differential expression analysis of astrocytoma cell lines with modulated GFAP-networks Volcano plots showing the –log10 of the FDR p-value of each gene plotted against the log2 fold change **(A-D)**. This visualizes the general effect of GFAPα^+^ (A), GFAPδ^+^ (B), GFAPpan^−^ (C), and GFAPα^−^ (D) on the transcriptome. Differentially expressed genes selected for analysis with GO are represented with red dots. Names of the genes with the highest fold change have been added in black, and names of genes in GO-clusters “ECM disassembly”, “ECM organization”, “ECM”, “ECM binding” and “Extracellular Region” in blue (A-D). Validation of the microarray results by qPCR of selected genes (*THNSL2, LINGO2, A2M, BASP1, OGDHL, PIEZO2, SOX11, PI15, GLUL, LAMA1, GAPDH, GFAPα, SULF1, CNOT10, PPP3CB, CLNS1A, SERP2, RAMP1*, and *CRYAB*) showed that the fold change assessment of microarray and qPCR were correlated, ρ=0.93 **(E)**. Clustering of the scaled expression changes revealed 4 main clusters of genes, dominated by parallel or opposite changes in expression of these genes between GFAPδ^+^ and GFAPα^−^, as indicated by different colors in the dendrogram (purple, gray, yellow, black) **(F)**.

To get insight into the function of these GFAP- isoforms in astrocytoma and to investigate a potential role for GFAPα and the GFAPδ/α ratio in astrocytoma malignancy, we modulated GFAP-isoform expression in U251-MG cells by recombinant expression or by silencing with shRNAs of the isoforms. We analysed the GFAP- isoform induced transcriptomic changes. We generated microarray-based whole-genome gene expression profiles of GFAPα^+^, GFAPδ^+^, GFAPpan^−^, and GFAPα^−^ astrocytoma cell lines. For validation of the cell lines and microarray data see Supplementary Figure 1. The results of the microarray are represented in volcano plots (Figure 2A-2D). Differentially expressed genes (FC>1.5, FDR<.1) are represented with red dots. GFAPα^+^ expression induced a differential expression of 57 genes and GFAPδ^+^ expression induced differential expression of 158 genes compared to control. GFAPpan^−^ and GFAPα^−^ cells were compared to the NTC. These resulted in 48 and 848 differentially expressed genes, respectively.

The array data were validated by qPCR. The following targets were selected to verify various fold changes and directions of change of expression patterns: *THNSL2, LINGO2, A2M, BASP1, OGDHL, PIEZO2, SOX11, PI15, GLUL, LAMA1, GAPDH, GFAPα, SULF1, CNOT10, PPP3CB, CLNS1A, SERP2, RAMP1*, and *CRYAB*. The original RNA samples that were used to generate the labelled cRNA probes for array hybridisation were used to make cDNA and analysed by qPCR (Figure 2E). The qPCR confirmed the findings of the microarray. Overall, the array and qPCR data highly correlated (ρ = 0.93, p-value<.001).

Genes with an FDR adjusted p-value of <.1 in both the recombinant expression and the knockdown models were clustered and evaluated in a heatmap. This analysis showed that GFAPα^−^ and GFAPδ^+^, which both had the largest impact on the GFAPδ/α ratio, had the highest contribution to the clustering of the heatmap. These two conditions led to four main emerging patterns, that either showed an effect in the same direction or in an opposite direction for GFAPα^−^ and GFAPδ^+^ (Figure 2F). The other experimental groups, GFAPpan^−^ and GFAPα^+^ had a lower effect on GFAPδ/α ratio, and had a less prominent effect on the clustering. The individual genes and their patterns are shown in Supplementary Figure 2. We conclude that not the change in GFAP per se, but the change in GFAPδ/α had the most pronounced effect on the transcriptome in astrocytoma cells.

The differentially expressed genes were tested for overrepresentation in a gene ontology analysis, to reveal potential functional differences related to the changes in the GFAP-network. This analysis of both the recombinant expression and knockdown cells resulted in a recurring overrepresentation of gene ontology clusters related to the cell periphery, such as “extracellular matrix”, “extracellular space” (everything extracellular except the matrix) or “plasma membrane” (Supplementary Tables 2-6). Remarkably, the 80 differentially expressed genes between GFAPδ^+^ and GFAPα^+^ were also overrepresented in the same clusters (Supplementary Table 6). These results show that both GFAP modulation per se, as well as modulation of the specific isoforms individually, affected genes involved in the composition of the extracellular matrix and the extracellular space. These findings corroborate our earlier work, where we showed that silencing *GFAPα* in astrocytoma cells led to a strong increase in *LAMA1* expression [25].

### *GFAPδ/α* ratio correlates to tumour malignancy and regulates high-malignant genes

To identify those GFAP-isoform induced transcriptomic changes that are relevant for astrocytoma malignancy, we compared our *in vitro* transcriptomic data to the TCGA derived patient transcriptomic data (Supplementary Figure 3A-3B). In patients, *GFAPα* expression and the *GFAPδ/α* ratio was significantly different between grade II and III versus grade IV astrocytoma (Figure 1). Therefore we compared transcriptomic changes induced by *in vitro* modulation of GFAPα (GFAPpan^−^, GFAPα^−^, GFAPα^+^) or the GFAPδ/α ratio (GFAPδ^+^, GFAPα^+^, GFAPα^−^) to transcriptomic differences between grade II and III versus grade IV astrocytoma (Supplementary Figure 3C).

**Figure 3:**
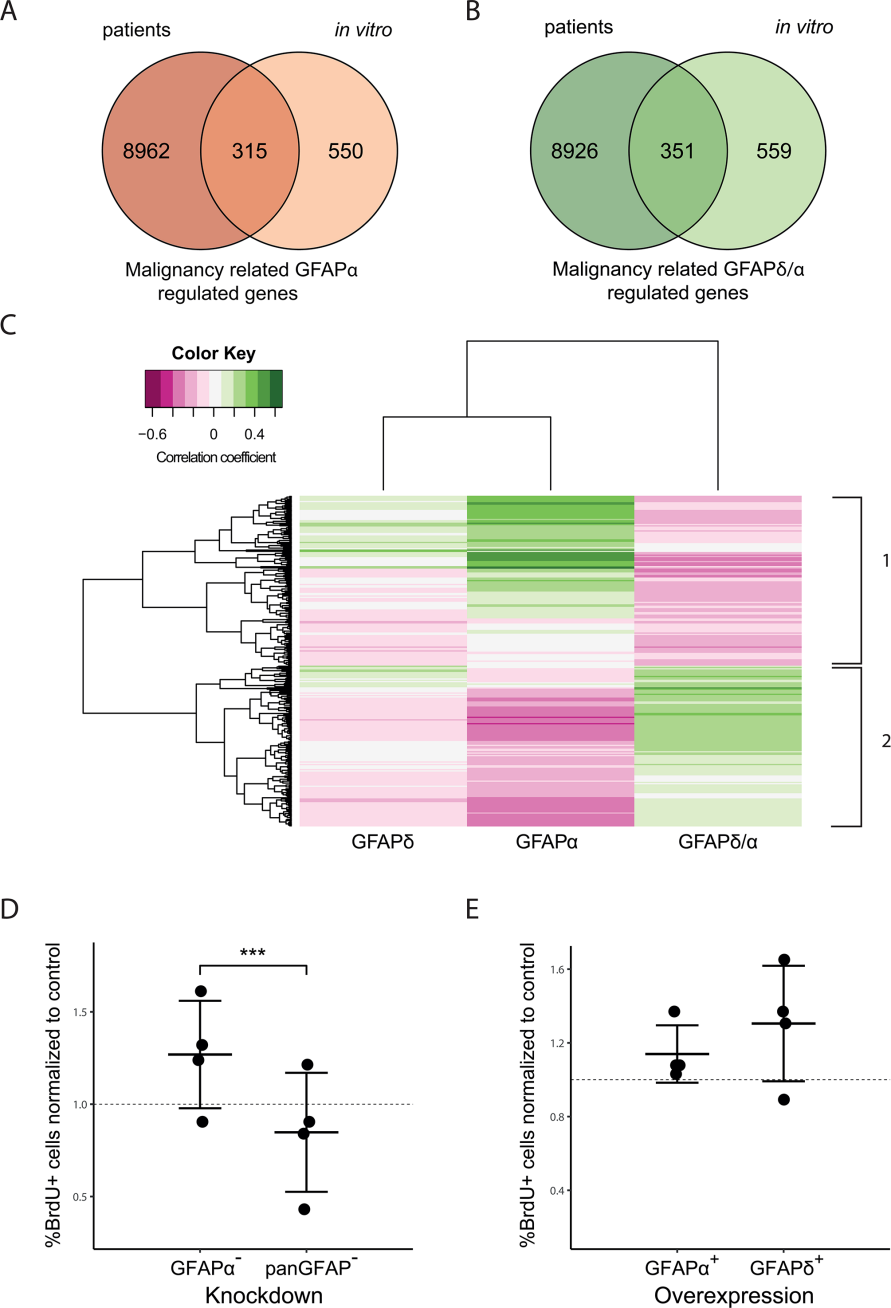
Comparison between GFAP-regulated genes in astrocytoma cells and patients Venn Diagrams show the overlap of genes differentially expressed between low and high grade astrocytoma in patients (left circles) and genes regulated by a change in the GFAP-network *in vitro* (right circles) **(A, B)**. 315 genes are both differentially expressed between astrocytoma of low and high grade as well as regulated by a change in GFAPα expression *in vitro* (A). 351 genes are both differentially expressed between astrocytoma of low and high grade as well as regulated *in vitro* by a change in the GFAPδ/α ratio (B). The intersect of both diagrams represent the genes identified as malignancy related GFAPα (315) or GFAPδ/α (351) regulated genes. For these genes, a linear regression analysis was performed. Correlation to *GFAPα* and *GFAPδ* expression, and the GFAPδ/α ratio in patients ranging from grade II to grade IV was determined. The heatmap shows the genes that significantly correlated to either *GFAPα* expression or the *GFAPδ/α* ratio (FDR < 0.01) **(C)**. The colour key indicates the correlation coefficient (ρ). Hierarchical clustering on the absolute correlation coefficient resulted in the identification of two main clusters. Cluster 1 consists of genes that positively correlate to *GFAPα* but negatively to the *GFAPδ/α* ratio, cluster 2 shows the opposite pattern with genes negatively correlating to *GFAPα* and positively to the *GFAPδ/α* ratio. A larger version of this figure is provided in Supplementary Figure 4. BrdU proliferation assay graphs **(D, E)** show the mean and standard deviation of the percentage of cells that incorporated BrdU as determined by immunocytochemistry. For each experiment (n=4) and condition, 5 images were analysed. The mean percentages of BrdU positive cells per condition and experiment were normalized to (D) NTC and (E) control. (D) GFAPα^−^ cells show a significant higher percentage of BrdU positive cells compared to GFAPpan^−^ cells (p=0.00017). (E) No difference was found in BrdU positive cell percentages between GFAPδ^+^ and GFAPα^+^ cells.

Figure 3A shows a Venn diagram for the comparison of genes regulated by a change in GFAPα *in vitro* and genes differentially expressed between astrocytoma of low and high grade. Of the 865 genes that were regulated by a change in GFAPα expression *in vitro* (FDR < 0.1, FC >1.5), 315 genes were differentially expressed between astrocytoma of low and high grade (FDR < 0.1, FC >1.5) and showed a similar direction of change as *GFAPα*. The comparison between genes regulated by a change in the GFAPδ/α ratio *in vitro* and differentially expressed genes between astrocytoma of low and high grade is visualized in Figure 3B. Of the 910 genes regulated by a change in GFAPδ/α, 351 were found to be differentially expressed between astrocytoma of low and high grade and showed a similar direction of change as the *GFAPδ/α* ratio.

We classified the overlapping genes between our *in vitro* and patient data set as malignancy related GFAPα-regulated genes or malignancy related GFAPδ/α-regulated genes. A linear regression analysis was performed on the overlapping genes (Supplementary Figure 3D), to test their correlation to *GFAPα*, *GFAPδ* and *GFAPδ/α* within grade II to IV astrocytoma. Genes that significantly correlated to either *GFAPα* or the *GFAPδ/α* ratio (FDR < 0.01) were clustered in a heatmap (Figure 3C). This hierarchical clustering resulted in the identification of two large gene clusters, i.e. genes that correlated positively to *GFAPα* and negatively to the *GFAPδ/α* ratio and genes that correlated negatively to *GFAPα* and positively to the *GFAPδ/α* ratio. These genes were classified as GFAP-regulated, since their expression level was also significantly regulated in the *in vitro* cells with modulated GFAP-networks. Based on their astrocytoma grade correlation, genes were further classified as high-malignant, i.e. higher expressed in grade IV compared to grade II and III, or low-malignant genes, i.e. lower expressed in grade IV compared to grade II and III (Supplementary Figure 3A).

We observed a positive correlation for *GFAPα*, a negative correlation for *GFAPδ/α* ratio, and no correlation for *GFAPδ* with astrocytoma grade (Figure 1). Therefore, we selected genes that showed a strong correlation to both *GFAPα* and *GFAPδ/α* ratio in opposite directions (FDR < 0.01), but did not correlate to *GFAPδ* (FDR > 0.1) as the most relevant high-malignant or low-malignant genes regulated by GFAP (Supplementary Figure 4 gives detailed information of the gene clusters in Figure 3C). This resulted in 43 genes that were identified as GFAP-regulated low-malignant genes due to their positive correlation to *GFAPα* and negative correlation to *GFAPδ/α* (Supplementary Table 7). The 37 genes that correlated negatively to *GFAPα* while they correlated positively to *GFAPδ/α* were identified as GFAP-regulated high-malignant genes (Supplementary Table 7).

### GFAP-regulated genes are involved in tumour biology

The GFAP-regulated high-malignant and low-malignant genes were tested for overrepresentation in a GO analysis to gain insight in the function of GFAPα and the GFAPδ/α ratio in astrocytoma (Supplementary Figure 3E). Table 1 shows significant GO clusters overrepresented by a minimum of 5 of these genes. Interestingly, three biological processes highly related to tumour malignancy-(‘mitotic cell cycle’, and ‘regulation of cell proliferation’ related to tumour growth and ‘regulation of phosphorylation’ involved in the activation or deactivation of many tumour malignancy related signalling pathways) were significantly overrepresented. As shown in Table 1, both GFAP-regulated low-malignant and high-malignant genes were overrepresented within these GO clusters. These results show that the GFAPα and GFAPδ/α ratio levels regulate cellular expression patterns of genes that are involved in regulating biological processes known to be different between astrocytoma of low and high grade. This supports a role for GFAP-isoforms and the GFAPδ/α ratio in astrocytoma malignancy.

**Table 1:** Overrepresented GO clusters by GFAP-isoform regulated high/low-malignant genes

GO ID	GO term	# sig gene	p-value	GFAP-isoform modulated low-malignant genes	GFAP-isoform modulated high-malignant genes
**Biological processes**					
GO:0000278	mitotic cell cycle	8	3.2E-4	TTYH1	SPC24, CENPP, CENPQ, PRIM1, RBBP8, RFC4, E2F8
GO:0042127	regulation of cell proliferation	9	0.020	EDNRB, SGK3, FBXO2, NTRK2, PURA, AKR1C3	VAV3, IGFBP5, NOS2
GO:0042325	regulation of phosphorylation	10	0.043	DMD, EDNRB, SASH1, SMAD7, NTRK2	VAV3, DUSP4, ECT2, BARD1, PDGFD
**Cellular Compartment**					
GO:0005829	cytosol	20	0.006	DMD, CYS1, SGK3, FBXO2, SMAD7, NTRK2, FMN2, MID1IP1, AHNAK, AKR1C3, GTPBP1	VAV3, POLR3G, IGF2BP3, SPC24, ECT2, CENPP, NOS2, CENPQ, DCTPP1
GO:0031090	organelle membrane	17	0.026	ST3GAL6, CPE, EDNRB, CYS1, TMEM59L, FBXO2, NTRK2, FMN2, SLC44A2, TTYH1, AHNAK, EFHD1	ECT2, MANEA, MYO19, PDGFD, HS3ST3B1
GO:0045121	membrane raft	7	5.6E-6	DMD, EDNRB, CYS1, FAIM2, MAL AHNAK	CXADR
**Molecular Function**					
GO:0042803	protein homodimerization activity	6	0.006	NTRK2, MYOM1	ECT2, NOS2, BARD1 E2F8

Genes identified as GFAP-isoform modulated high/low malignant genes were analysed for gene ontology overrepresentation, in the Biological Processes, Cellular Compartment and Molecular Function domain.

GO ID: Gene ontology identifier, # sig gene: Number of significant genes in the GO term.

### GFAP-isoform modulation alters astrocytoma cell proliferation *in vitro*

In order to confirm the role of GFAP-isoforms and the GFAPδ/α ratio in mitosis and cell proliferation as was suggested by the GO analysis, we performed an *in vitro* Bromodeoxyuridine (BrdU) proliferation assay using the GFAP-modulated astrocytoma cell lines. We analyzed BrdU incorporation of GFAPpan^−^ and GFAPα^−^ cells and normalized the percentage of BrdU positive cells to the NTC (Figure 3D). We found a significant higher % BrdU positive cells in GFAPα^−^ astrocytoma cells compared to GFAPpan^−^ indicative of a higher proliferation rate in GFAPα^−^ cells. Analysis of BrdU incorporation of GFAPδ^+^ and GFAPα^+^ cells did not show any differences in proliferation rate between cells (Figure 3E). These results furthermore support a role of the GFAPδ/α ratio in cell mitosis and proliferation and show that an increase in the GFAPδ/α ratio (GFAPα^−^ compared to GFAPpan^−^) resulted in increased proliferation rates and is therefore associated with a higher malignant phenotype.

### GFAP-regulated high-malignant and low-malignant genes are associated with patient survival

**Figure 4:**
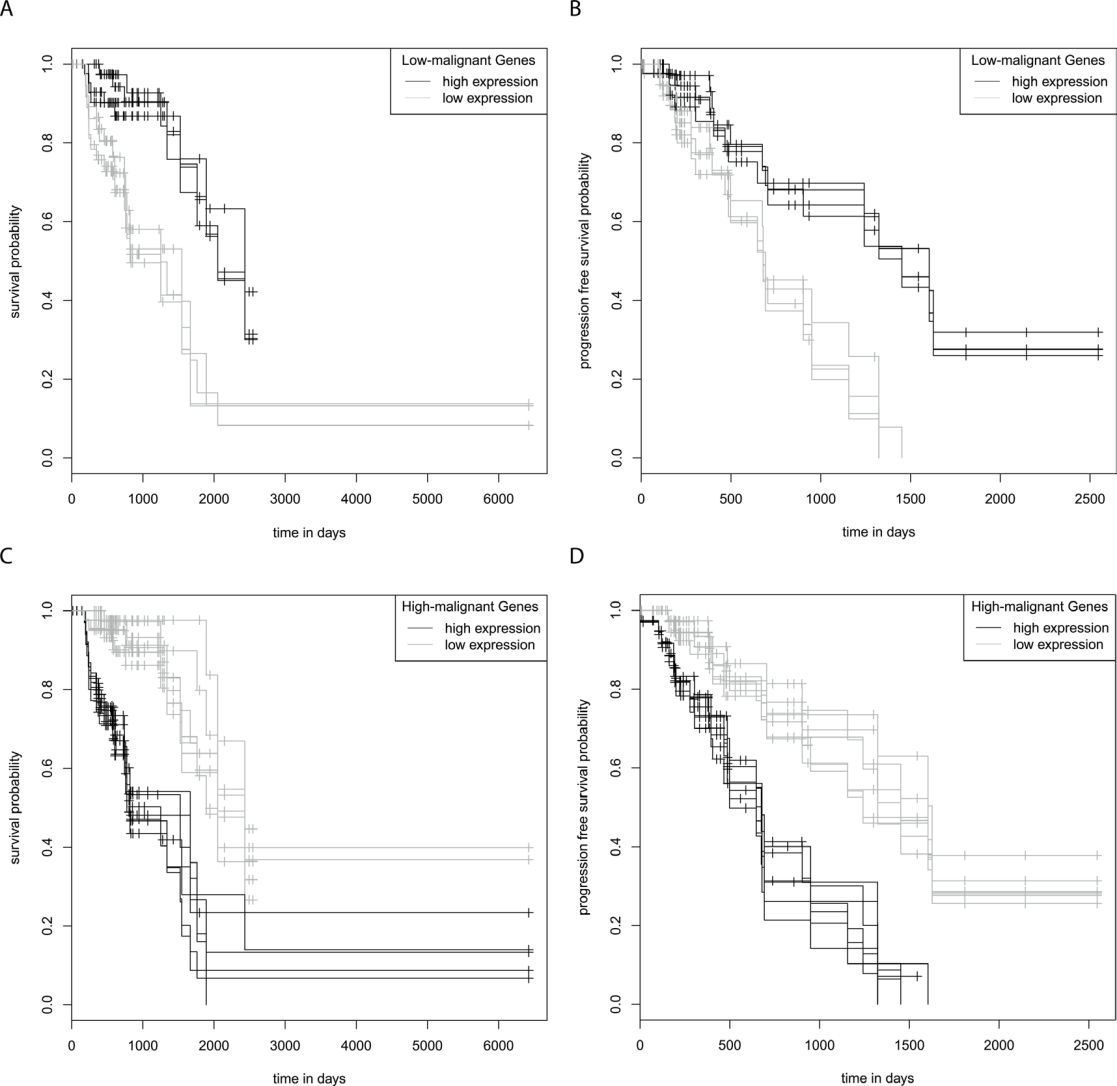
Kaplan-Meier survival and progression free survival curves grade III astrocytoma The graphs show survival and progression free survival curves of below and above median expression of GFAP-regulated low-malignant **(A, B)** and high-malignant genes **(C, D)**. Survival and progression free survival curves are shown for genes of which a high expression predicts both a significantly shorter survival and a progression free survival compared to low expression, or vice versa (FDR < 0.05). Low expression of the low-malignant genes *AKR1C3, ARHGEF10L, SMAD7* and *ST3GAL6* predicts a significant shorter survival (A) and progression free survival (B) High expression of the high-malignant genes *C7ORF46, DUSP4, HS3T3B1, ODZ1, TTL, VAV3* and *ZDHHC23* predicts a significant shorter survival (C) and progression free survival (D).

In order to test whether differences in GFAPα and GFAPδ/α levels contribute to patient outcome, we performed Kaplan-Meier analyses for patient survival within each astrocytoma grade patient group (Supplementary Figure 3E). No significant differences were found between survival and progression free survival estimates for grade II, III, or IV astrocytoma patients with below (low) or above (high) median *GFAPα* expression or a below or above median *GFAPδ/α* ratio. These results indicate that *GFAPα* and the *GFAPδ/α* ratio per se do not directly modulate tumour characteristics that affect survival of patients within one astrocytoma grade. However, the survival estimates for below and above median expression of the GFAP-regulated high-malignant or low-malignant genes were significantly different for 32 of these genes in astrocytoma grade III patients. Interestingly, high expression of the high-malignant genes and low expression of the low-malignant genes was associated with a lower survival probability (FDR < 0.05) (Supplementary Table 8). Moreover, high expression of 7 of the high-malignant genes (*C7ORF46, DUSP4, HS3ST3B1, ODZ1, TTL, VAV3, ZDHHC23*) or low expression of 4 of the low-malignant genes (*AKR1C3, ARHGEF10L, SMAD7, ST3GAL6*) was associated with a lower progression free survival probability of grade III astrocytoma patients (Figure 4A-4D).

**Figure 5:**
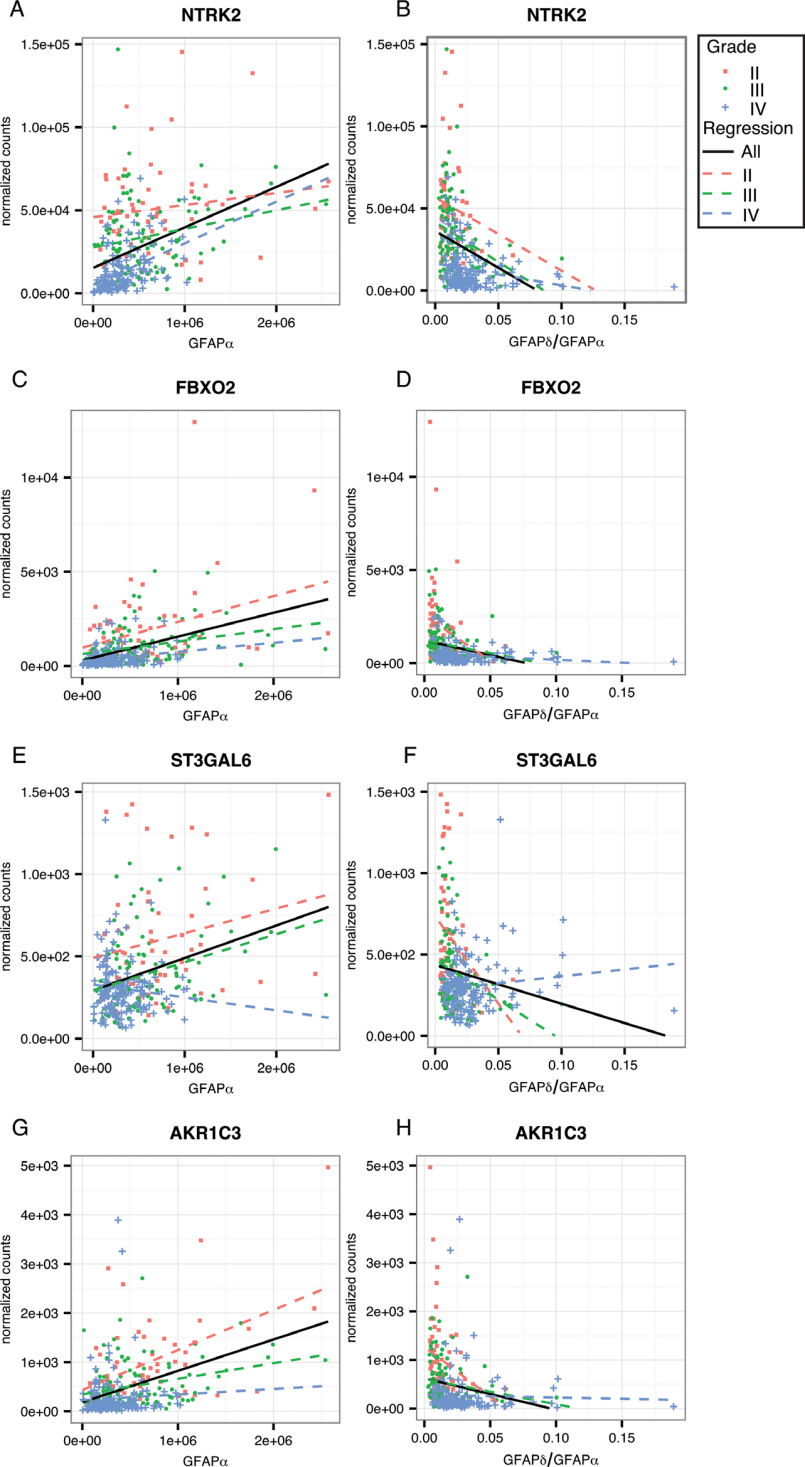
Linear regression analysis within astrocytoma grades for GFAP-regulated low-malignant genes Scatterplots show the distribution of *GFAPα* normalized isoform expression and the *GFAPδ/α* ratio (=*GFAPδ/GFAPα* normalized isoform expression) plotted against the normalized gene expression of each gene of interest. The black line shows the regression line for a correlation when all patients are included in the analysis. Red squares depict astrocytoma grade II patients, green dots astrocytoma grade III, and blue crosses depict astrocytoma grade IV patients. Dotted lines show the regression line for a correlation within grade II (red), grade III (green), and grade IV (blue) astrocytoma. **(A-H)** GFAP-regulated low-malignant genes that significantly correlated to *GFAPα* or *GFAPδ/α* (FDR < 0.05) in either grade II, III or IV, and were related to patient survival and progression free survival (*AKR1C3, ST3GAL6*) and/or were present in one of the three significantly overrepresented GO Biological Processes clusters (*NTRK2, FBXO2, AKR1C3*) are shown here. The results of the linear regression analysis are reported in Supplementary Table 7.

**Figure 6:**
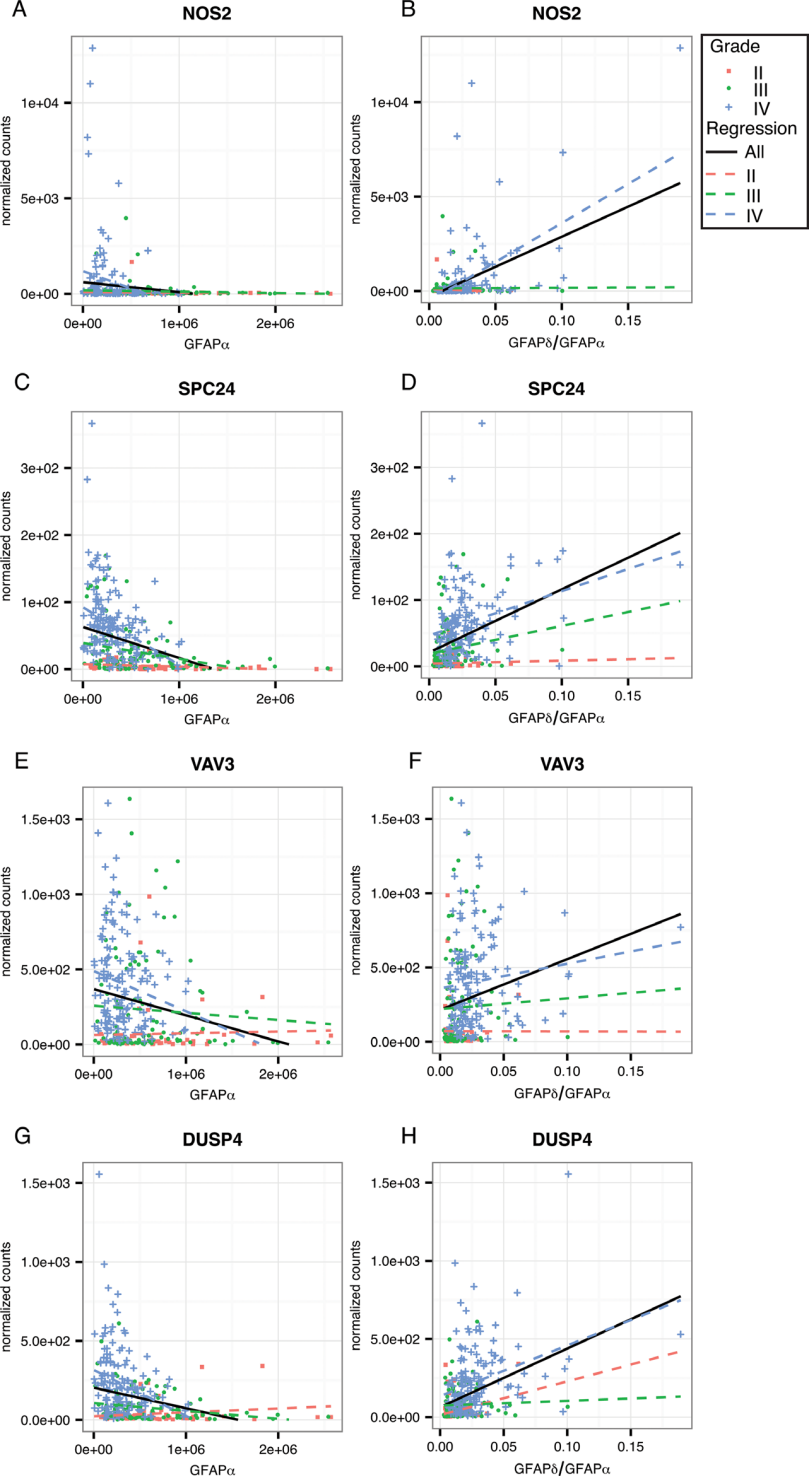
Linear regression analysis within astrocytoma grade for GFAP-regulated high-malignant genes Scatterplots show the distribution of *GFAPα* normalized isoform expression and the *GFAPδ/α* ratio (=*GFAPδ/GFAPα* normalized isoform expression) plotted against the normalized gene expression of each gene of interest. The black line shows the regression line for a correlation when all patients are included in the analysis. Red squares depict astrocytoma grade II patients, green dots astrocytoma grade III, and blue crosses depict astrocytoma grade IV patients. Dotted lines show the regression line for a correlation within grade II (red), grade III (green) and grade IV (blue) astrocytoma. **(A-H)** GFAP-regulated high-malignant genes that significantly correlated to *GFAPα* or *GFAPδ/α* (FDR < 0.05) in either grade II, III or IV, and were related to patient survival (*VAV3, DUSP4*) and/or were present in one of the three significantly overrepresented GO Biological Processes clusters (DUSP4, SPC24, VAV3, NOS2) are shown here. The results of the linear regression analysis are reported in Supplementary Table 7.

To further investigate the regulation of these genes of interest by GFAP, we calculated the strength of correlation to *GFAPα* and the *GFAPδ/α* ratio within astrocytoma grade (Supplementary Figure 3F). This reduces the influence of astrocytoma grades on the correlation of GFAP with expression levels of a gene. For this analysis, we only considered genes that were overrepresented in one of the gene ontology clusters or that were associated with a worse progression free survival probability. We found that 4 of the genes that we identified as GFAP-regulated high-malignant genes: i.e. *NTRK2, FBXO2, ST3GAL6, and AKR1C3*, also correlated with GFAPα or GFAPδ/α ratio within an astrocytoma grade (Figure 5A-5H, corresponding statistics in Supplementary Table 9). The set of genes identified as GFAP-regulated low-malignant genes, also contained 4 genes which showed the GFAP-dependent pattern within one or more of the astrocytoma grades (*NOS2, SPC24, VAV3*, and *DUSP4*; Figure 6A-6H, corresponding statistics in Supplementary Table 9).

The genes that emerged from this final analysis are the genes we consider most likely to be regulated by a change in GFAP-isoforms in astrocytoma. We show that the regulation of their expression could influence astrocytoma malignancy through alterations in biological processes highly involved in the regulation of tumour malignancy (mitosis, proliferation, phosphorylation). In addition, regulation of these genes by a change in GFAP-isoforms could alter the biology of the tumour in such a way that it influences disease progression and survival of astrocytoma grade III patients.

## DISCUSSION

Intermediate filament proteins, such as vimentin and some keratins, emerge as important regulators in various malignancies [11, 14]. GFAP is the signature IF of astrocytoma cells, nevertheless the function of GFAP in astrocytoma biology is still unclear. In order to gain new insights into astrocytoma biology, we studied *GFAP*-isoform expression in resected astrocytoma from patients (TCGA database) as well as the downstream transcriptional changes in astrocytoma cells caused by modulation of these GFAP-isoforms. With this approach we identified low-malignant and high-malignant genes that are regulated by GFAP. These genes regulate cellular processes highly related to tumour malignancy as apparent from our GO analysis, and the expression levels of these genes have a prognostic value for astrocytoma grade III patients.

Our earlier work showed that an experimentally induced shift in the GFAPδ/α ratio leads to changes in cellular motility and morphology [24, 25]. Therefore, we hypothesized that the stoichiometry of the GFAP-isoforms determines specific molecular and functional changes in astrocytoma cells related to their malignancy. Indeed the most pronounced transcriptional changes in our study were induced by the largest increase in *GFAPδ/α* ratio. More importantly, the increase in this ratio correlated with an increase in astrocytoma malignancy. Although GFAP immunoreactivity is decreased with increasing astrocytoma grade [17], it is also known that a complete lack of all GFAP-isoforms, as in the GFAP^−/−^ mice, is not sufficient to increase tumorigenicity [33]. We here show that it is the ratio between different GFAP-isoforms, and not the mere expression level of GFAP, that contributes to malignancy. In fact, a positive correlation between GFAPδ and the degree of astrocytoma malignancy was described earlier in a small number of patients, however in these patients GFAPα was not evaluated [21–23].

### GFAP-regulated high/low-malignant genes in tumour biology

From our current study we identified GFAP-regulated high-malignant genes, i.e. *VAV3* (a guanine exchange factor for Rho GTPases), *NOS2* (a nitrous oxide synthase), *DUSP4* (a phosphatase of e.g. ERK) and *SPC24* (part of the kinetochore complex), and GFAP-regulated low-malignant genes, i.e. *NTRK2* (a tyrosine kinase receptor for e.g. BDNF), *FBXO2* (a ubiquitin ligase), *ST3GAL6* (a sialyltransferase for glycosylated proteins) and *AKR1C3* (an aldo-keto reductase involved in prostaglandin metabolism). These genes were regulated by the GFAPδ/α ratio *in vitro* and correlated to this ratio in astrocytoma patients (Figure 5 and 6). Our classification of these genes as high-malignant or low-malignant is based on their correlation to astrocytoma malignancy and is supported by previous studies. Increased expression of VAV3 in high compared to low grade astrocytoma [34, 35], increased NOS2 expression in tumours of higher compared to lower malignancy and in brain tumours compared to healthy tissue [36–39], and increased SPC24 expression in other tumour types compared to healthy tissue [40] all support their tumorigenic function. This is emphasized by the negative prognostic effect of VAV3 and NOS2 expression in grade IV astrocytoma and for SPC24 in liver tumours [40]. Similarly, decreased NTRK2 expression in high compared to low grade astrocytoma [41], decreased ST3GAL6 expression in liver tumours [42], and increased AKR1C3 expression in 1q19p co-deleted glioma compared to other glioma subtypes [43] support a low-malignant function, although other studies on NTRK2 [44, 45], AKR1C3 [46, 47], and ST3GAL6 [48] are inconsistent.

Our gene ontology analysis indicates that the GFAPδ/α ratio controls astrocytoma malignancy by regulating tumour growth. This is supported by the increase in cell proliferation induced by the increased GFAPδ/α ratio in astrocytoma (Figure 3D-3E). Previous studies indeed report a functional role of our identified GFAP-regulated gene set in the regulation of proliferation, mitosis, and tumour growth. While the high-malignant gene *SPC24* positively regulates cell proliferation [40, 49], the low-malignant gene FBXO2 induces growth arrest [50, 51]. NOS2 and VAV3 stimulate glioma-initiating cell proliferation and tumour growth in xenograft models [34, 52].

Invasive capacity is another important factor in tumour malignancy. Previous studies have suggested a role of the IF-network in astrocytoma cell invasion and interaction with the ECM [24, 25]. For brain tumours to grow and invade the brain, ECM remodelling is an essential step [53]. Interestingly, GO analysis on all GFAP-isoform induced transcriptional changes observed in our astrocytoma cell lines revealed the cell's interaction with its environment through adhesion, ECM composition, and ECM remodelling as the major targeted modules regulated by GFAP modulation (Supplementary Tables 2-6). Remarkably, the 80 differentially expressed genes between GFAPδ^+^ and GFAPα^+^ are also overrepresented in the same clusters representing interaction with the environment (Supplementary Table 6) suggesting a differential role of the isoforms in these processes. In addition, an Ingenuity Pathway Analysis of annotations and function in physiology and pathology with a focus on cancer, indicated a significant role of GFAP-isoform regulated genes in tumour invasion (*data not shown*). Moreover, our identified GFAP-regulated high-malignant and low-malignant genes are involved in invasion [34, 35, 39, 40, 54, 55]. Focal adhesion formation is essential for cell migration and invasion and our previous studies indicated that changes in the expression of GFAP-isoforms regulate expression of plectin and integrins, genes involved in focal adhesion formation [25] and the size of focal adhesions in astrocytoma cell lines [24]. In addition, the IF protein vimentin, VAV2, and Rac1 interact in the regulation of the invasive capacity of lung cancer cells by controlling the formation of focal adhesions via FAK [16]. Such an interaction between focal adhesion components and IFs is a recurring theme in IF biology [56] and as the guanine-nucleotide exchange factor VAV3 modulates invasion probably by targeting the Rho-GTPase Rac [35, 57, 58] a similar interaction between GFAP, VAV3 and Rho-GTPases in focal adhesions can be hypothesized. Interestingly, *NOS2*, a target of DUSP4, and *DUSP4* [59], both factors that stimulate tumour migration and invasion [34, 35, 39], are regulated by GFAP. This suggests that changes in the GFAPδ/α ratio affects multiple components of signalling pathways.

The relationship between GFAP-isoforms and astrocytoma malignancy that we show here is based on a correlation. However, this correlation is supported by the causal relationship between GFAP-isoform modulation and the expression of low- and high-malignant genes and proliferation *in vitro*. We modulated the GFAP- network by down regulation and by overexpression of the GFAP-isoforms. As becomes apparent from our BrdU experiments, changing the GFAPδ/α ratio by down regulation or overexpression leads to different outcomes. Both a high level of GFAPδ in the cell by recombinant expression, which is not observed under physiological conditions, and a subtle decrease in GFAPα using shRNA result in a higher GFAPδ/α ratio. However, these changes have different effects on the composition of the GFAP-network: recombinant expression of GFAPδ results in a collapse of the network. Such a severe effect on the GFAP-network will not always occur with a knock down of GFAPα. Thus, the absolute expression levels and the precise stoichiometry of the GFAP-isoforms will determine the cell's behaviour. Therefore, future in depth studies that focus on these isoform specific alterations, its effect on the GFAP-network, and the malignant behaviour of the cell are needed. Nevertheless, we here provide evidence for an important role of GFAP-isoforms in astrocytoma malignancy that will direct future research.

Together, our data and previous observations suggest that GFAP-isoforms in astrocytoma regulate the malignant phenotype of cells both by regulating their growth as well as their invasive capacity. We here showed that the stoichiometry of GFAP-isoforms modulates expression of genes involved in the cell's interaction with its environment and the ECM.

In conclusion, our data supports an important role for the IF-network in astrocytoma malignancy. Astrocytomas of high grade have different network compositions induced by an increased ratio of *GFAPδ/GFAPα*, leading to changed expression of genes that are involved in tumour growth and invasion and are associated with survival and progression of disease in astrocytoma grade III patients. We identified *VAV3*, *NOS2*, *DUSP4*, and *SPC24* as high-malignant, and *NTRK2*, *FBXO2*, *ST3GAL6*, and *AKR1C3* as low-malignant genes that are all regulated by the GFAP-network. Thus, GFAP-isoforms have an important function in astrocytoma malignancy and the GFAP-network and *GFAP* alternative splicing are novel targets that await further exploration as a potential treatment for astrocytoma.

## MATERIALS AND METHODS

### Sample selection from the Cancer Genome Atlas

TCGA contains data of 528 astrocytoma grade IV cases (also known as GBM) and 516 lower grade glioma cases. Of the latter group 168 were classified as astrocytoma grade II or grade III cases. RNA sequencing data was available for 165 GBM and all grade II and III cases. We excluded recurrent tumours from this study and averaged the results of the analysis on the same tumour samples (same vial). The final cohort, included in our analysis, consisted of 55 grade II, 105 grade III, and 150 grade IV astrocytoma samples (Table 2).

**Table 2:** Clinical characteristics of included astrocytoma patients

Astrocytoma	Grade II	Grade III	Grade IV
**N (number of included patients)**	55	105	150
***N survival analysis***	41	91	150
**Age, years**			
***Median (LQ, UQ)***	36 (30, 66)	42 (34, 74)	60 (52, 89)
***Average***	37.16	44.17	59.61
**Survival, days**			
***Median (LQ, UQ)***	230 (114.5, 3283)	300 (155, 6423)	280.5 (146.5, 1642)
***Censored, %***	94.64	77.36	34.67
**Karnofsky score**			
***100***	13	16	12
***90***	8	27	2
***70-80***	8	18	67
***<70***	2	5	30
***NA***	24	39	39
**Gender**			
***Male***	31	61	97
***Female***	24	44	53

Age, survival, Karnofsky score, and gender specifications of included patients as provided by the TCGA database. LQ: lower quartile, UQ: upper quartile, NA: not available, TCGA: The Cancer Genome Atlas.

### RNA sequencing data and statistics

An explorative differential gene expression analysis between astrocytoma patients of different grade was performed in R software for statistical computing using the limma package in bioconductor (http://www.bioconductor.org). Expression data of grade II, III, and IV patients containing normalized gene and RNA isoform expression levels were obtained from TCGA data portal (Level 3 released data downloaded June, 2015), and expression levels were extracted as upper quartile normalized RSEM (RNA-Seq by Expectation Maximization) count estimates (normalized counts). Statistical significance was tested using a Bayesian linear model fit with an FDR corrected criterion level of α =.1. Genes were considered differentially expressed if the test reached statistical significance and showed at least a 1.5 fold absolute change. Normalized isoform expression level 3 data was used to determine *GFAPα* and *GFAPδ* expression levels and for each patient sample, the *GFAPδ/α* ratio was calculated. Statistical difference in the *GFAPδ/α* ratio and the *GFAPδ* or *GFAPα* expression level between grade II, III, and IV was tested using the non-parametric independent 2-group Mann-Whitney U Test, since the criteria for normal distribution were not met (Shapiro-Wilk test). The p-values were corrected with an FDR correction.

### GFAP-isoform modulation in astrocytoma cell lines

To explore the transcriptional changes induced by GFAP-isoform levels in astrocytoma, we performed *in vitro* GFAP-isoform modulation experiments. For these experiments we used two subclones of the human astrocytoma cell line U251-MG. One subclone had a lower endogenous level of GFAP and was used to study the effect of inducing GFAPα and GFAPδ expression. The other subclone had a higher endogenous level of GFAP and was used to knock down specific GFAP-isoforms and modulate the ratio between the different isoforms. U251-MG cells were transduced with lentiviral vectors containing recombinant-mCherry (referred to as “control”), recombinant human GFAPα-IRES-eGFP (referred to as “GFAPα^+^”) or recombinant human GFAPδ-IRES-mCherry (referred to as “GFAPδ^+^”) as described before [24]. These cells were maintained in 1:1 DMEM GlutaMAX high glucose: Ham's F-10 nutrient mix, supplemented with 100 U/ml penicillin, 100 μg/ml streptomycin (1% P/S) and 10% (v/v) FBS (all Invitrogen, Bleiswijk NL). The subclone with higher endogenous GFAP was transduced with lentiviral vectors containing shRNAs targeting either *GFAPα* (referred to as “GFAPα^−^”; shRNA targeted to the 3′UTR in exon 9), *GFAPpan* (referred to as “GFAPpan^−^”, shRNA targeted exon 2 which is present in all known GFAP-isoforms), or with a non-targeting control shRNA construct (referred to as “NTC”) as described by Moeton et al [25]. These cells were maintained in DMEM GlutaMAX high glucose supplemented with 1% P/S and 10% FBS. Transduction efficiency was maintained by culturing cells with puromycin (2 μg/ml). Cell identity was confirmed by short terminal repeat analysis.

The serum concentration in the cell culture medium was changed from 10% to 2% FBS three days before an experiment. For the microarray analysis, GFAPα^+^ and GFAPδ^+^ cells or GFAPα^−^ or GFAPpan^−^ cells were plated in medium with 2% FBS in 6-well flexible bottom Bioflex plates coated with the laminin derived peptide YIGSR (Flexcell International, Hillsborough NC, US; Dunn Labortechnik, Asbach, DE), at a density of 5×10^4^ cells/cm^2^. For maintenance, all cells were split twice a week and cultured in uncoated tissue culture plastics at 37°C, under a humidified 5% CO_2_/95% air atmosphere.

### RNA isolation

RNA was isolated by adding TRIsure (Bioline, London, UK; GC biotech B.V., Alphen aan den Rijn, NL) to the cell culture wells. RNA was extracted according to the manufacturer's protocol, and precipitated in isopropyl alcohol in the presence of glycogen at −20°C overnight (Roche; Almere) [60]. Samples were centrifuged at 4°C and at a speed of 2.0^*^10^4^ g for 45 minutes (min), washed twice with cold 75% ethanol (in MilliQ (Millipore)), and the RNA pellets were dissolved in MilliQ. The 260/280 absorbance ratio was measured by spectrophotometry (NanoDrop; Thermo Scientific) to assess RNA concentration and purity.

### Real time quantitative PCR

qPCR was performed as described before [60]. For extensive description, see Supplementary Materials. Primer pairs used are reported in Supplementary Table 1.

### RNA labelling, hybridization, and scanning

cRNA was made from 100 ng RNA using a Low Input Quick Amp Labelling Kit, two-colour (Agilent, Amstelveen, NL), as per manufacturer's protocol. A mix of 825 ng of Cy3 and Cy5 labelled cRNA samples was hybridized to Human GE 4×44K v2 Microarrays (Agilent, Amstelveen, NL) according to the manufacturer's protocol. The recombinant expression samples (N=8 per condition: control, GFAPα^+^, GFAPδ^+^) and the GFAP KD samples (N=5 for GFAPα^−^, N=5 for GFAPpan^−^, N=6 for NTC) were hybridized to the microarrays. cRNA fluorescence intensity was scanned using an Agilent DNA Microarray Scanner. Scans were made with 100% and 10% photon multiplier tube intensity, at 5 μm resolution. The microarray data have been submitted to the Gene Expression Omnibus (GSE74567).

### Microarray data processing

Data processing was performed as described before [61, 62]. Briefly, data were extracted from microarray images using Feature Extraction software. Using the limma package in bioconductor (http://www.bioconductor.org), spot intensities were imported into R software for statistical computing. Spots labelled by the Feature Extraction software as saturated or non-uniformly distributed foregrounds and backgrounds, as well as visually identified artefacts on the array were omitted. In these cases the statistical analysis of differential expression was based on the remaining spots. Intensities were quantile normalized between all arrays, and sample intensities were extracted for an intensity-based analysis [63]. Redundant probes detecting identical transcripts were averaged. All genes that did not have a condition with an average hybridisation intensity of log_2_ > 6 were omitted from the analysis.

### Microarray statistics

Statistical significance was tested using a Bayesian linear model fit with an FDR corrected criterion level of α =.1 to prevent type II errors [64]. The analysis of overexpression and knockdown cells were performed independently of one another. For recombinant expression, all three conditions (control (N=8), GFAPα^+^ (N=8), and GFAPδ^+^ (N=8)) were compared. For knockdown, contrasts between the two KD conditions (GFAPα^−^ (N=5), GFAPpan^−^ (N=5)) versus NTC (N=6) were made. Microarray probes were considered to report a differentially expressed gene if the statistical test was significant and the fold change showed at least a 1.5 fold absolute change.

### Linear regression analysis

*In vitro* differentially expressed genes (our microarray data) induced by changes in GFAP-isoforms (FDR <0.1; FC > 1.5) were compared to transcriptomic (TCGA data) differences between astrocytoma of different grade (FDR <0.1; FC > 1.5). A comparison was made between *in vitro* GFAPα regulated genes (observed in the GFAPα^−^, GFAPpan^−^, GFAPα^+^ conditions) and differentially expressed genes between grade II and grade IV, and grade III and grade IV, analysed as described above. The same comparison was made for *in vitro* GFAPδ/α ratio regulated genes (observed in the GFAPδ^+^, GFAPα^−^, and GFAPα^+^ conditions). The TCGA gene expression levels of the overlapping genes were tested for a significant correlation to *GFAPα*, *GFAPδ*, and *GFAPδ/α* ratio in patients by fitting a linear regression model to the data. P-values were FDR adjusted. At FDR < 0.01 genes were identified as strongly correlating genes. Gene expression of the final set of high/low-malignant genes was correlated to *GFAPα*, *GFAPδ* and *GFAPδ/α* ratio within each astrocytoma grade in the same way. Correlation was considered significant at FDR < 0.05.

### Gene ontology

To detect GO clusters that were overrepresented in our datasets we used the topGO R package [65]. We used a Fisher statistical test to test for overrepresentation, with a weighted algorithm to correct for dependency between parent-child relations between ontology clusters [66]. The test for overrepresentation of our genes of interest was performed in the context of all Entrez gene IDs considered in the statistical test for differential expression on the RNAseq data. Databases used to search for overrepresentation were the GO domains ‘Cellular compartment’ (CC), ‘Biological Process’ (BP) and ‘Molecular Function’ (MF). Annotation was performed using an annotation build of 10-Aug-2015 (org.Hs.eg.db, ID = entrez) for the analysis on our RNAseq data set.

### Survival analysis

To test whether survival and progression free survival estimates for patients with below or above median expression of a gene of interest were significantly different, we calculated survival and progression free survival probabilities with a Kaplan-Meier survival analysis using the Survival package in R. Survival and progression free survival data were available for 41 grade II and 91 grade III astrocytoma patients. Survival data was available for 150 grade IV astrocytoma patients (downloaded from TCGA January, 2016). Estimates were compared using the log rank regression analysis and were considered different at an FDR adjusted significance level of 0.05. A trend for a difference between estimates was determined at FDR < 0.1.

### BrdU proliferation assay

To determine the role of the GFAPδ/α ratio in cell mitosis and proliferation we performed a Bromodeoxyuridine (BrdU) proliferation assay on the GFAP modulated astrocytoma cell lines (GFAPα^+^, GFAPδ^+^ and control cells or GFAPα^−^, GFAPpan^−^ and NTC cells). Cells were plated on poly-D lysine coated (20 μg/ml) cover slips at a density of 20.000 cells/well in a 24 well plate in culture medium containing 2% FBS. Cells were exposed to 0% FBS containing medium overnight after which the medium was changed to 10% containing medium for 4 hours. Subsequently, cells were treated with 40 nM BrdU for 2 hours, then they were fixed with 4% (w/v) paraformaldehyde (4% PFA) dissolved in phosphate buffer saline (PBS, pH 7.4) for 30 min. Cells were washed in PBS and antigens were retrieved by incubation for 30 min in citrate buffer (10 mM citric acid, pH 6.0 and 0.05% Tween-20) in a steamer. Cells were incubated O/N at 4°C with anti-BrdU (mouse monoclonal, Dako, Denmark) in Tris-buffered saline (TBS, pH 7.6). Cells were washed in TBS and incubated for 1 hour in donkey-anti-mouse Cy3/488 (Jackson Immuno Research, USA) and Hoechst (33528, Thermo Fisher Scientific, USA) to counterstain the nuclei. Cells were washed in TBS, dipped in MilliQ and mounted with Mowiol (0.1 M tris-Hcl pH 8.5, 25% glycerol, 10% Mowiol (Calbiochem, Merck Millipore, Darmstadt, Germany)). Images were taken with a 10x objective using a Zeiss Axioscope.A1 microscope. Per condition, 5 images were analysed and the mean percentage of BrdU positive nuclei was calculated using ImageJ and normalized to the controls (knockdown: N=4, overexpression: N=4). A Shapiro-Wilk test was done to test for normal distribution of the data and a paired t-test was performed to test for significant differences between the normalized means.

## SUPPLEMENTARY MATERIALS FIGURES AND TABLES






