# Supplementary material for: GFAPδ/GFAPα ratio directs astrocytoma gene expression towards a more malignant profile
Source: Oncotarget. 2017 Oct 4;8(50):88104–21. doi: 10.18632/oncotarget.21540 (PMC5675697; doi:10.18632/oncotarget.21540)
Supplement: Supplementary file 1 [file oncotarget-08-88104-s001.pdf]

GFAPδ/GFAPα ratio directs astrocytoma gene expression towards a more malignant profile

SUPPLEMENTARY MATERIALS

Real time quantitative PCR

After treating 500 ng RNA with DNAse for 2 min at 42°C (gDNA wipeout buffer), cDNA was made in 10 µl reactions using Quantiscript® Reverse Transcriptase with a mix of oligo-dT and random primers at 42°C for 30 min. The reaction was stopped by an inactivation step at 95°C for 3 min (Quantitect; Qiagen Benelux B.V., Venlo, NL).

The produced cDNA was diluted 20x in MilliQ and was used as a template in the real time qPCR in 96-wells plates and the reaction was analysed with an ABI7300 (Applied Biosystems, Life Technologies, Bleiswijk, NL). The reaction mix consisted of 1 µl template, 3.5 µl MilliQ, 0.5 µl primer mix (final concentration of 0.1 µM for each primer), and 5 µl SYBR® Green PCR Master Mix (Applied Biosystems, Life Technologies, Bleiswijk, NL). The qPCR program consisted of an incubation of 2 min at 50°C and 10 min at 95°C, followed by 40 cycles of 15 seconds at 95°C and 1 min at 60°C. After the qPCR reaction, a dissociation curve was made by ramping the temperature from 60°C to 95°C. Curve analysis was performed using the Sequence Detection Software version 1.4 (Applied Biosystems), with a standard threshold of 0.2 (determined to be in the log linear part of the derived curve), and an automatic determination and correction of baseline fluorescence. Primers were designed to target intron-spanning exons (when possible) of the transcripts of interest and to generate 50-125 bp long amplicons. Primer efficiencies and dissociation curves were verified using a 1:20-1:320 dilution series of U251-MG cDNA and the amplicon product size was verified by agarose gel electrophoresis. Specifications of primers are given in Supplementary Table 1. A set of 3 reference genes was identified based on the microarray analysis (PPP3CB, CNOT10, CLNS1A), of which the geometric mean was used to normalize the data.

Immunocytochemistry

For immunocytochemical stainings, cells were fixed in 4% (w/v) paraformaldehyde (4% PFA) dissolved in phosphate buffered saline, pH 7.4 (PBS) for 15 min. Cells were washed with PBS and incubated overnight at 4°C with the primary antibody diluted in blocking buffer (50 mM Tris pH 7.4, 150 mM NaCl, 0.25% (w/v) gelatine, and 0.5% triton X-100). Cells were then washed three times with PBS and incubated with the secondary antibodies and the nuclear counterstain Hoechst 33258, diluted in blocking buffer, at room temperature for 1 hour. Cells were then washed three times with PBS, dipped in MilliQ and mounted with Mowiol (0.1 M tris-Hcl pH 8.5, 25% glycerol, 10% Mowiol (Calbiochem, Merck Millipore, Darmstadt, Germany)). Micrographs from the fluorescent stainings were taken with a Leica epifluorescent DMRD microscope. The antibody used was polyclonal anti-GFAP (1:2000; DAKO, #Z0334) and the nuclei were counterstained with Hoechst 33258 (1:1000; invitrogen).

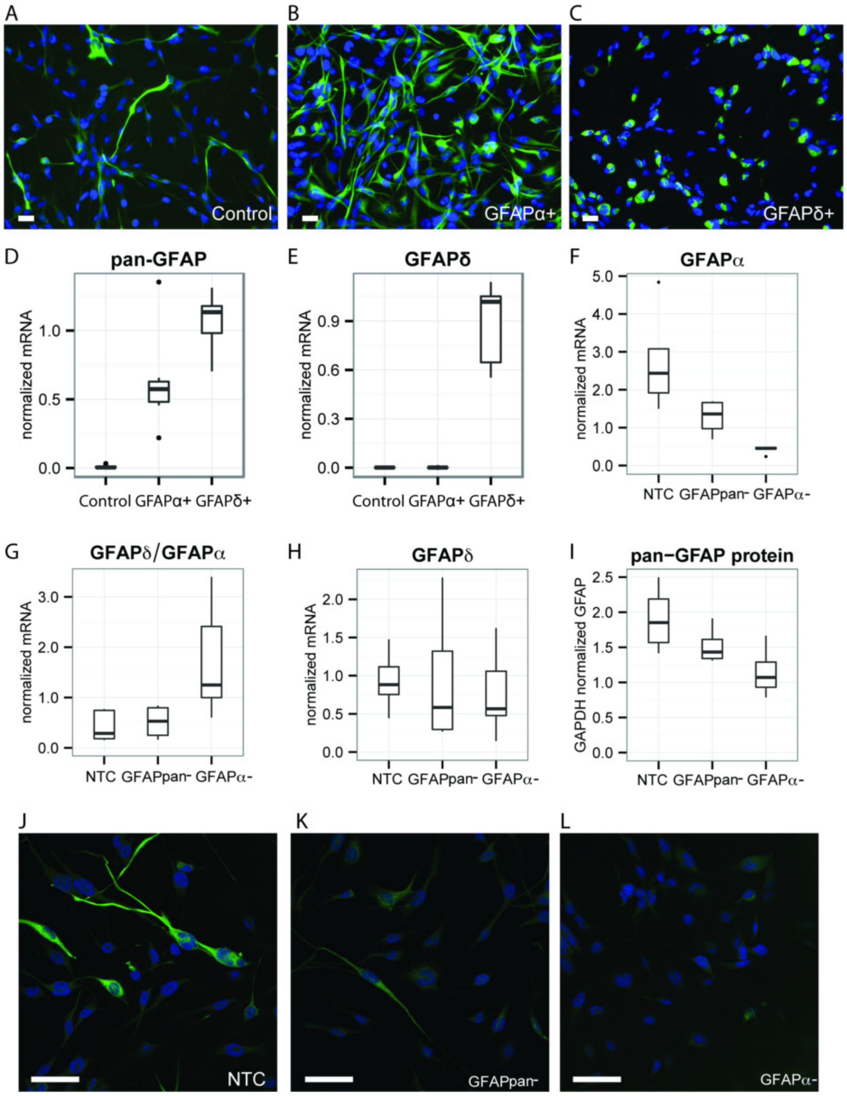

Supplementary Figure 1: Modulation of GFAP-network in U251 astrocytoma cells. To get insight into the function of these GFAP-isoforms in astrocytoma and to investigate a potential role for GFAPα and the GFAPδ/α ratio in astrocytoma malignancy, we modulated GFAP-isoform expression in U251 cells by recombinant expression **(A-E)** or silencing with shRNAs **(F-L)** of the isoforms and analysed the GFAP-isoform induced transcriptomic changes. Cells were transduced with *GFAPα* (GFAPα$^+$) or *GFAPδ* (GFAPδ$^+$). Staining the control cells showed some cells positive for endogenous GFAP, but predominantly GFAP-negative cells (A) GFAPα$^+$ cells showed a GFAP-network throughout the cytoplasm (B), whereas GFAPδ$^+$ cells showed the characteristic juxtanuclear accumulation of GFAP (C), as we have described before [1]. At the RNA level, using primers that recognize all (pan) *GFAP*-isoforms, a significant increase of pan-*GFAP* expression in GFAPα$^+$ (FC = 9.6, FDR<.001) and GFAPδ$^+$ (FC = 16.3, FDR<.001) cells was observed (D). *GFAPδ* mRNA was only increased in GFAPδ$^+$ cells (FC = 1534, FDR<.001) (E). Depicted is the median (horizontal line) with quartiles (box), N=8. These finding are in agreement with western blot results showing that the amount of GFAP protein is increased in these cell lines as shown before [1]. Cells were transduced with shRNA directed against pan-*GFAP* (GFAPpan$^-$) or against *GFAPα* (GFAPα$^-$)**.** Both the GFAPpan$^-$ (FC = 0.46, FDR=.063) and the GFAPα$^-$ (FC = 0.15, FDR=.027) cells showed a decrease in *GFAPα* mRNA (F). GFAPα$^-$ cells shifted their ratio of *GFAPδ/α* mRNA more towards *GFAPδ*, due to a strong decrease in *GFAPα* (ratio of *GFAPδ/α:* the ratio for NTC = 0.42; GFAPpan$^-$ = 0.51, FDR=.31; GFAPα$^-$ = 1.73, FDR=.077) (G), while the *GFAPδ* was stable (H) N=5, depicted is the median (horizontal line) with quartiles (box)). The knockdown of GFAP was confirmed by western blot, and was more efficient in the GFAPα$^-$ than in the GFAPpan$^-$ cells (I) depicted is median with quartiles (box), N=4. Stainings confirmed the less efficient knockdown of GFAP in the GFAPpan$^-$ (K) than the GFAPα$^-$ (L), compared to NTC cells (J). Scale bars in the micrographs represent 50 um.

References

1. Moeton M, Stassen OM, Sluijs JA, van der Meer VW, Kluivers LJ, van Hoorn H, Schmidt T, Reits EA, van Strien ME, Hol EM. GFAP isoforms control intermediate filament network dynamics, cell morphology, and focal adhesions. Cell Mol Life Sci. 2016; 73:4101–4120. doi: 10.1007/s00018-016-2239-5.

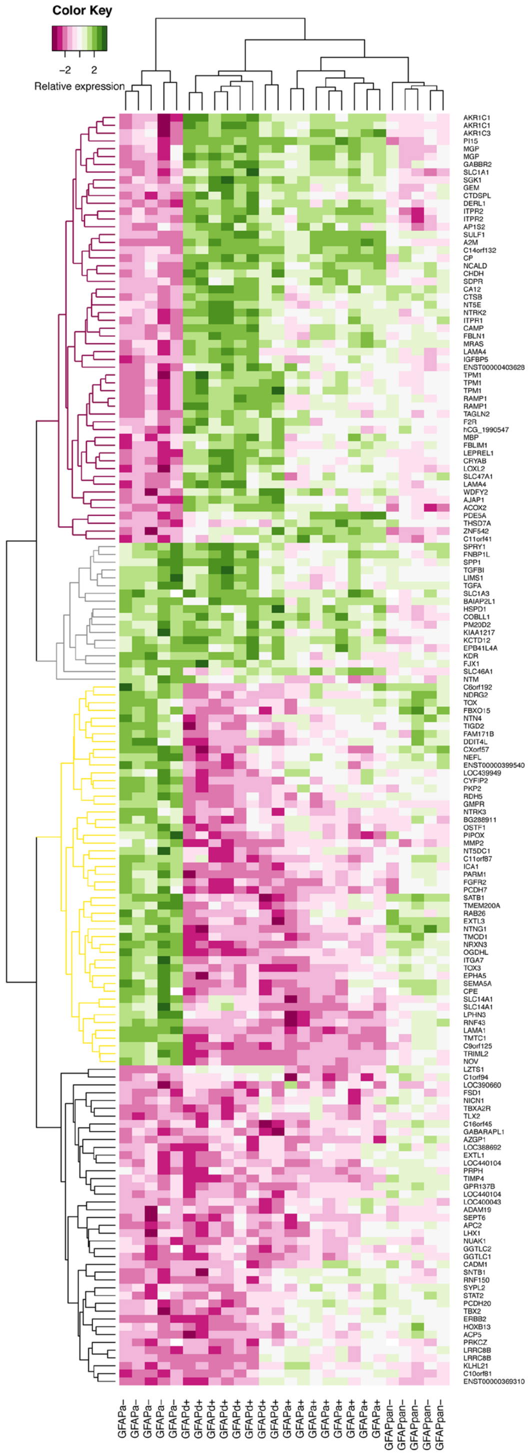

Supplementary Figure 2: Heat map of genes with an FDR p-value <. 1 in at least one knockdown and one recombinant expression condition. Four main patterns were distinguished of either upregulation in both GFAPα^{-} and GFAPδ^{+} or downregulation in both GFAPα^{-} and GFAPδ^{+}, or an opposite regulation by GFAPα^{-} and GFAPδ^{+}, as indicated by different colors in the dendrogram (purple, grey, yellow, black). Some genes showed similar behaviour in GFAPα^{+} as well as in GFAPδ^{+} cells, whereas GFAPpan^{-} in general didn't show large effects. The observed strongest changes in the GFAPα^{-} and GFAPδ^{+} condition imply that the change is due to the increase in GFAPδ/GFAPα ratio.

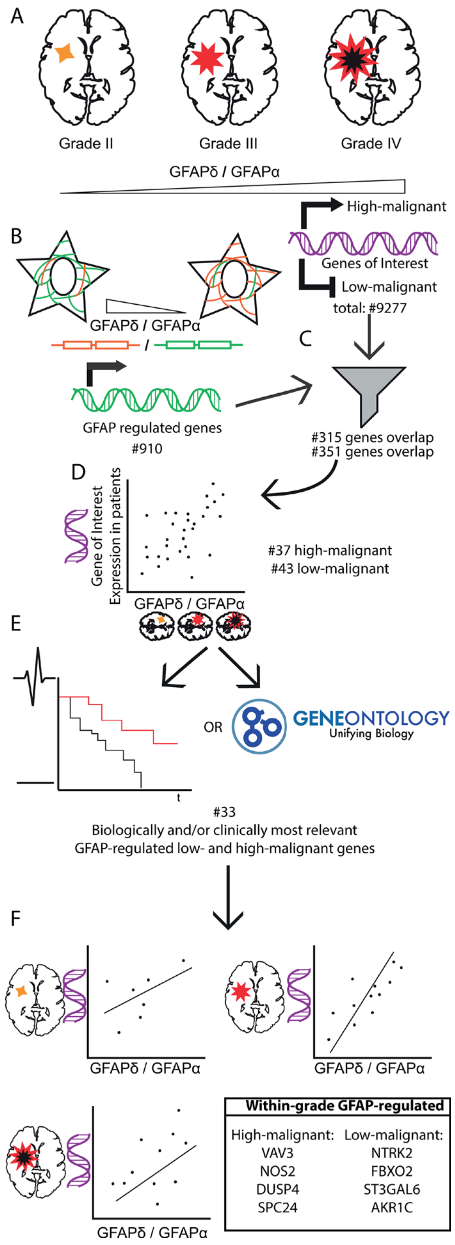

Supplementary Figure 3: Processing steps of the data in this study. After finding that *GFAPα* and *GFAPδ/GFAPα* ratio correlate with astrocytoma grade (Figure 1), all genes that were differentially expressed across astrocytoma grades in patients were identified from the TCGA database. **(A)** To select genes that are regulated by GFAPα and GFAPδ/GFAPα ratio, we modulated the GFAP-network in astrocytoma cell lines *in vitro* and identified differentially expressed genes regulated by GFAP. **(B)** The cell line dataset was used to filter the astrocytoma data. **(C)** From these combined patient/*in vitro* genes we calculated initial correlations between GFAP-isoforms/GFAP-ratio and target genes in the patient data and identified a set of GFAP-regulated high-malignant and low-malignant genes. These sets are GFAP-regulated based on their *in vitro* regulation by GFAP-isoforms. Gene sets are high-malignant or low-malignant based on their expression pattern in astrocytoma patients. **(D)** To identify the subset of genes relevant for astrocytoma progression and biology, the filtered genes were analyzed for survival, progression free survival, and gene ontology. **(E)** To further reduce the risk of confounding by astrocytoma grade, a linear regression was performed between the most interesting GFAP-regulated low- and high malignant genes and *GFAP* expression within the different astrocytoma grades. **(F)** Finally, 8 genes passed all these criteria and emerged as the most probable GFAP-regulated low- and high malignant genes with biological or clinical relevance in astrocytoma.

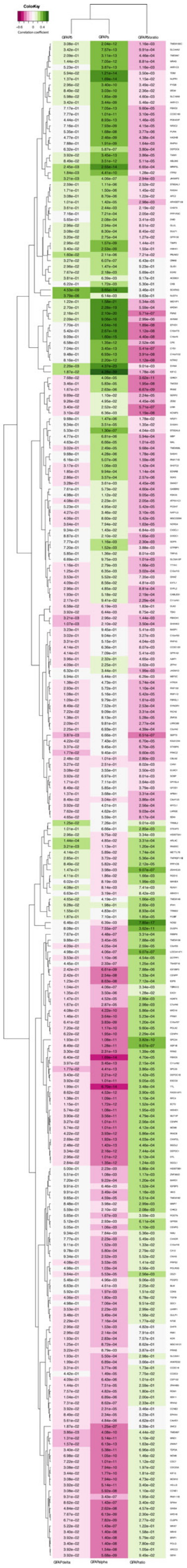

Supplementary Figure 4: Heatmap of the genes that significantly correlated to either *GFAPα* or the *GFAPδ/α* ratio (FDR < 0.01). The color key indicates the correlation coefficient (R). Hierarchical clustering on the absolute correlation coefficient results into the identification of two main clusters. Cluster 1 consists of genes that positively correlate to *GFAPα* but negative to the *GFAPδ/α* ratio, cluster 2 shows the opposite pattern with genes negatively correlating to *GFAPα* and positively to the *GFAPδ/α* ratio.

Supplementary Table 1: Primer pairs used and their qPCR efficiencies for microarray validation

Genename	E-value	Amplicon size	Sequence FW 5'-> 3'	Sequence RV 5'-> 3'
ALU-SQ [1]	1.85	variable	CATGGTGAAACCCCGTCTCTA	GCCTCAGCCTCCCGAGTAG
ALU-J [2]	1.9	variable	CAACATAGTGAAACCCCGTCTCT	GCCTCAGCCTCCCGAGTAG
LAMA1	1.98	120	GTTTCGAACCTCCTCGCAGA	CTGCCAGCACCATTGTTGAC
BASP1	2	59	GCTAACTCAGGGGCTGCATA	TGAGCTTGCCTCCCATCTTG
PI15	1.94	112	GGCGACGTGCAGTTTACTTG	CCCCCATAACTTGGAGGACA
OGDHL	2	120	CTGGATCGTGGTCAACTGCT	GGGTGCCTCAGCAGAGATTT
A2M	2	83	CTGAGTACAATGCTCCTTGCAG	AACAGGACTCCAGCAAAGCA
RAMP1	1.92	64	CAGGGACGTGACCTTGACTTA	TGGCTATGGGGTAAGCAGTC
GFAPpan	2	50	GCCACCTACAGGAAGCTGCT	GGGAATGGTGATCCGGTTCT
GFAPα (endogenous)	1.95	114	CCCACTCTGCTTTGACTGAGC	CCTTCTTCGGCCTTAGAGGG
GFAPδ (endogenous & recombinant)	1.92	96	TCCAACCTGCAGATTCGAGG	TTGGTATAACTCGTATTGTGAGGCTT
PPP3CB	2	116	CTGCAGCCCGGAAAGAAATCA	AGTCAGGCCCTTGAGTGTCA
CNOT10	1.9	110	ACAGCAAGATCCAAAGCAGGA	TGCAAGCAAGTATGGAGCAC
CLNS1A	1.85	108	AGTTGCTGGACAGTTTGAGGA	AGAGTGGAAGAGGCACCAAG
THNSL2	1.9	125	TGGCAGTGAACCGCAATGAC	CCTCTCCATGTTGTAGGGCAC
LINGO2	2[a]	104	CGAAGGAGAATTAACAGCGCC	AACATGGATGCTCCAACTTCA
SOX11	2	54	TCCGGTTTGGGACTCCTAGT	TGGCCGTCACGAAGCATTAT
CRYAB	1.89	76	TTCCCCACCAGTGAATGAAAG	CTTGATAATTTGGGCCTGCC
SERP2	1.95	96	TGGCTAACGAGAAGCACAGC	GCCATGGTCCCACAGGATATT
SULF1	2	120	CACTGTCCGAGTGACACACA	TGCCTTATGGTCCTTCCACG
GAPDH	2	87	TGCACCACCAACTGCTTAGC	GGCATGGACTGTGGTCATGA
GLUL	2	94	TGTGTGGAAGAGTTGCCTGAG	TGGCAGCAGGCACGAGATAC

E-value: Efficiency defined as fold increase of amplicon per qPCR cycle, [a]no product on calibration curve: 2 used for calculations, size: amplicon size in basepairs, FW:forward primer; RV: reverse primer.

REFERENCES

1. Rihani A, Van Maerken T, Pattyn F, Van Peer G, Beckers A, De Brouwer S, Kumps C, Mets E, Van der Meulen J, Rondou P, Leonelli C, Mestdagh P, Speleman F, Vandesompele J. Effective Alu repeat based RT-Qpcr normalization in cancer cell perturbation experiments. PLoS One. 2013; 8:e71776. doi: 10.1371/journal.pone.0071776.

2. Witt N, Rodger G, Vandesompele J, Benes V, Zumla A, Rook GA, Huggett JF. An assessment of air as a source of DNA contamination encountered when performing PCR. J Biomol Tech. 2009; 20:236–240.

Supplementary Table 2: Top Gene Ontology terms per domain overrepresented by GFAPα[+] compared to control

GO domain	GO.ID	Term	Annotated genes	Sig genes	p-val
BP	GO:0090162	establishment of epithelial cell polarity	11	2	8.6E-04
BP	GO:0030513	positive regulation of BMP signalling pathway	14	2	1.4E-03
BP	GO:0048706	embryonic skeletal system development	79	4	2.9E-03
BP	GO:0003338	metanephros morphogenesis	17	2	4.0E-03
BP	GO:0002011	morphogenesis of an epithelial sheet	24	2	4.2E-03
BP	GO:0007566	embryo implantation	29	2	6.1E-03
BP	GO:0007218	neuropeptide signalling pathway	35	2	8.8E-03
BP	GO:0021762	substantia nigra development	38	2	1.0E-02
BP	GO:0008016	regulation of heart contraction	83	3	1.1E-02
BP	GO:0030323	respiratory tube development	114	3	1.2E-02
CC	GO:0005887	integral component of plasma membrane	600	9	4.8E-04
CC	GO:0005615	extracellular space	547	9	2.2E-03
CC	GO:0005576	extracellular region	2022	16	6.2E-03
CC	GO:0008180	COP9 signalosome	31	2	6.6E-03
CC	GO:0031012	extracellular matrix	225	3	1.0E-02
CC	GO:0005796	Golgi lumen	39	2	1.0E-02
CC	GO:0072562	blood microparticle	59	2	2.3E-02
MF	GO:0030414	peptidase inhibitor activity	67	2	7.6E-03
MF	GO:0005507	copper ion binding	38	2	9.5E-03

Annotated genes = number of total microarray probes annotated to this term; Sig genes = number of probes detecting significant change in this term; p-val = parent-child weighted p-value of Fisher’s exact test for overrepresentation; GO = Gene Ontology; BP = Biological Process; CC = Cellular Component; MF = Molecular Function.

Supplementary Table 3: Top Gene Ontology terms per domain overrepresented by GFAPδ^{+} compared to control

GO domain	GO.ID	Term	Annotated genes	Sig genes	p-val
BP	GO:0030198	extracellular matrix organization	241	17	6.3E-06
BP	GO:0016264	gap junction assembly	5	3	1.2E-05
BP	GO:0007267	cell-cell signalling	619	25	3.1E-05
BP	GO:0072224	metanephric glomerulus development	8	3	6.8E-05
BP	GO:0022617	extracellular matrix disassembly	73	6	1.4E-04
BP	GO:0090162	establishment of epithelial cell polarity	11	3	1.9E-04
BP	GO:0033081	regulation of T cell differentiation in thymus	12	3	2.6E-04
BP	GO:0030539	male genitalia development	13	3	3.3E-04
BP	GO:0007616	long-term memory	14	3	4.2E-04
BP	GO:0001666	response to hypoxia	172	7	4.8E-04
CC	GO:0005615	extracellular space	548	23	5.0E-06
CC	GO:0005887	integral component of plasma membrane	601	18	1.3E-04
CC	GO:0005886	plasma membrane	2364	57	2.6E-04
CC	GO:0072562	blood microparticle	59	5	5.1E-04
CC	GO:0030667	secretory granule membrane	36	4	6.8E-04
CC	GO:0031095	platelet dense tubular network membrane	6	2	1.8E-03
CC	GO:0005922	connexon complex	6	2	1.8E-03
CC	GO:0031988	membrane-bounded vesicle	1623	26	2.3E-03
CC	GO:0009897	external side of plasma membrane	98	6	2.3E-03
CC	GO:0030054	cell junction	522	17	8.6E-03
MF	GO:0005021	vascular endothelial growth factor-activated receptor activity	5	2	1.2E-03
MF	GO:0005243	gap junction channel activity	7	2	2.4E-03
MF	GO:0051879	Hsp90 protein binding	12	2	7.2E-03
MF	GO:0005246	calcium channel regulator activity	13	2	8.5E-03
MF	GO:0017080	sodium channel regulator activity	14	2	9.8E-03
MF	GO:0050839	cell adhesion molecule binding	46	3	1.4E-02
MF	GO:0038023	signalling receptor activity	439	13	1.4E-02
MF	GO:0016709	oxidoreductase activity, acting on paired donors, with incorporation or reduction of molecular oxygen, NAD(P)H as one donor, and incorporation of one atom of oxygen	19	2	1.8E-02
MF	GO:0015459	potassium channel regulator activity	20	2	2.0E-02
MF	GO:0030414	peptidase inhibitor activity	68	4	2.1E-02

Annotated genes = number of total microarray probes annotated to this term; Sig genes = number of probes detecting significant change in this term; p-val = parent-child weighted p-value of Fisher's exact test for overrepresentation; GO = Gene Ontology; BP = Biological Process; CC = Cellular Component; MF = Molecular Function.

Supplementary Table 4: Top Gene Ontology terms per domain overrepresented by GFAPpan^{-} compared to NTC

GO domain	GO.ID	Term	Annotated genes	Sig genes	p-val
BP	GO:0090036	regulation of protein kinase C signalling	14	2	1.0E-03
BP	GO:0014910	regulation of smooth muscle cell migration	23	2	2.8E-03
BP	GO:0014068	positive regulation of phosphatidylinositol 3-kinase signalling	35	2	6.5E-03
BP	GO:0048146	positive regulation of fibroblast proliferation	36	2	6.9E-03
BP	GO:0045740	positive regulation of DNA replication	45	2	1.1E-02
BP	GO:0016192	vesicle-mediated transport	821	6	1.2E-02
CC	GO:0005902	microvillus	48	2	1.1E-02
CC	GO:0016021	integral component of membrane	3172	17	3.2E-02
CC	GO:0005576	extracellular region	2262	14	3.4E-02
MF	GO:0004177	aminopeptidase activity	29	2	4.1E-03

Annotated genes = number of total microarray probes annotated to this term; Sig genes = number of probes detecting significant change in this term; p-val = parent-child weighted p-value of Fisher's exact test for overrepresentation; GO = Gene Ontology; BP = Biological Process; CC = Cellular Component; MF = Molecular Function.

Supplementary Table 5: Top Gene Ontology terms per domain overrepresented by GFAPα⁻ compared to NTC

GO domain	GO.ID	Term	Annotated genes	Sig genes	p-val
BP	GO:0001525	angiogenesis	273	42	2.4E-07
BP	GO:0030198	extracellular matrix organization	276	41	1.5E-06
BP	GO:0007399	nervous system development	1432	131	8.2E-06
BP	GO:0043406	positive regulation of MAP kinase activity	150	15	2.2E-05
BP	GO:0050679	positive regulation of epithelial cell proliferation	105	17	1.1E-04
BP	GO:0048102	autophagic cell death	6	4	1.2E-04
BP	GO:0001570	vasculogenesis	53	11	1.6E-04
BP	GO:0007155	cell adhesion	661	73	2.5E-04
BP	GO:0008406	gonad development	145	25	2.5E-04
BP	GO:0060445	branching involved in salivary gland morphogenesis	19	8	2.7E-04
CC	GO:0005886	plasma membrane	2713	222	1.8E-05
CC	GO:0005604	basement membrane	73	14	3.4E-05
CC	GO:0005887	integral component of plasma membrane	708	67	4.4E-05
CC	GO:0042383	sarcolemma	68	14	3.0E-04
CC	GO:0016021	integral component of membrane	3219	231	5.3E-04
CC	GO:0005615	extracellular space	637	57	5.6E-04
CC	GO:0043679	axon terminus	48	8	1.2E-03
CC	GO:0009986	cell surface	332	33	1.5E-03
CC	GO:0031012	extracellular matrix	269	42	1.7E-03
CC	GO:0042995	cell projection	1021	69	2.6E-03
MF	GO:0016595	glutamate binding	7	4	2.7E-04
MF	GO:0008201	heparin binding	89	14	3.0E-04
MF	GO:0050840	extracellular matrix binding	36	8	8.9E-04
MF	GO:0042813	Wnt-activated receptor activity	16	5	1.3E-03
MF	GO:0000293	ferric-chelate reductase activity	5	3	1.5E-03
MF	GO:0004954	prostanoid receptor activity	5	3	1.5E-03
MF	GO:0005007	fibroblast growth factor-activated receptor activity	5	3	1.5E-03
MF	GO:0001968	fibronectin binding	17	5	1.7E-03
MF	GO:0005178	integrin binding	64	10	2.3E-03
MF	GO:0008022	protein C-terminus binding	146	17	2.5E-03

Annotated genes = number of total microarray probes annotated to this term; Sig genes = number of probes detecting significant change in this term; p-val = parent-child weighted p-value of Fisher's exact test for overrepresentation; GO = Gene Ontology; BP = Biological Process; CC = Cellular Component; MF = Molecular Function.

Supplementary Table 6: Top Gene Ontology terms per domain overrepresented by comparing GFAPα⁺ to GFAPδ⁺

GO category	GO.ID	Term	Annotated genes	Significant genes	p-val
BP	GO:0001525	angiogenesis	251	7	1.8E-04
BP	GO:0060291	long-term synaptic potentiation	20	3	2.0E-04
BP	GO:0016264	gap junction assembly	5	2	3.3E-04
BP	GO:0030198	extracellular matrix organization	242	8	3.7E-04
BP	GO:0045109	intermediate filament organization	7	2	6.9E-04
BP	GO:2000378	negative regulation of reactive oxygen species metabolic process	10	2	1.5E-03
BP	GO:0007616	long-term memory	14	2	2.9E-03
BP	GO:0051965	positive regulation of synapse assembly	16	2	3.8E-03
BP	GO:0048662	negative regulation of smooth muscle cell proliferation	16	2	3.8E-03
BP	GO:0007612	learning	64	3	6.1E-03
CC	GO:0005615	extracellular space	548	14	6.3E-05
CC	GO:0031988	membrane-bounded vesicle	1623	11	6.8E-04
CC	GO:0072562	blood microparticle	59	3	4.7E-03
CC	GO:0043195	terminal bouton	24	2	8.3E-03
CC	GO:0005886	plasma membrane	2366	26	1.7E-02
CC	GO:0005576	extracellular region	2023	26	1.9E-02
CC	GO:0009986	cell surface	283	5	2.1E-02
CC	GO:0005887	integral component of plasma membrane	602	8	2.2E-02
CC	GO:0005882	intermediate filament	87	3	2.9E-02
MF	GO:0008201	heparin binding	73	3	8.4E-03
MF	GO:0005125	cytokine activity	80	3	1.1E-02
MF	GO:0043169	cation binding	2757	18	1.4E-02
MF	GO:0030170	pyridoxal phosphate binding	39	2	2.1E-02

Annotated genes = Number of total microarray probes annotated to this term; Sig genes = number of probes detecting significant change in this term; p-val = parent-child weighted p-value of Fisher's exact test for overrepresentation; GO = Gene Ontology; BP = Biological Process; CC = Cellular Component; MF = Molecular Function.

Supplementary Table 7: Linear regression results of the 43 identified low-malignant genes and 37 identified high-malignant genes

See Supplementary File 1.

Supplementary Table 8: Kaplan Meier survival analysis of GFAP-regulated high- and low-malignant genes in grade III astrocytoma patients

Gene Name	p-value	FDR	HM/LM	Direction of expression and survival probability
DUSP4	4.13E-07	3.30E-05	HM	high expression, lower survival probability
VAV3	1.35E-06	5.40E-05	HM	high expression, lower survival probability
E2F8	1.46E-05	3.51E-04	HM	high expression, lower survival probability
ZDHHC23	1.76E-05	3.51E-04	HM	high expression, lower survival probability
MANEA	2.00E-04	3.20E-03	HM	high expression, lower survival probability
AKR1C3	2.83E-04	3.78E-03	LM	low expression, lower survival probability
HADHB	1.22E-03	9.69E-03	LM	low expression, lower survival probability
SMAD7	1.19E-03	9.69E-03	LM	low expression, lower survival probability
TMEM59L	1.06E-03	9.69E-03	LM	low expression, lower survival probability
ECT2	1.33E-03	9.69E-03	HM	high expression, lower survival probability
IGF2BP3	1.21E-03	9.69E-03	HM	high expression, lower survival probability
C14orf132	1.88E-03	1.03E-02	LM	low expression, lower survival probability
BARD1	1.66E-03	1.03E-02	HM	high expression, lower survival probability
HS3ST3B1	1.84E-03	1.03E-02	HM	high expression, lower survival probability
TTL	1.93E-03	1.03E-02	HM	high expression, lower survival probability
ODZ1	2.25E-03	1.13E-02	HM	high expression, lower survival probability
ARHGEF10L	2.89E-03	1.36E-02	LM	low expression, lower survival probability
ST3GAL6	4.55E-03	1.82E-02	LM	low expression, lower survival probability
C7orf46	4.44E-03	1.82E-02	HM	high expression, lower survival probability
TMEM158	4.17E-03	1.82E-02	HM	high expression, lower survival probability
CYS1	6.18E-03	2.36E-02	LM	low expression, lower survival probability
C10orf75	6.68E-03	2.43E-02	LM	low expression, lower survival probability
CXADR	8.95E-03	3.11E-02	HM	high expression, lower survival probability
LOC541471	1.06E-02	3.52E-02	HM	high expression, lower survival probability
TMEM182	1.26E-02	4.03E-02	HM	high expression, lower survival probability
C5orf35	1.32E-02	4.06E-02	HM	high expression, lower survival probability
PDGFD	1.44E-02	4.27E-02	HM	high expression, lower survival probability
SPC24	1.52E-02	4.34E-02	HM	high expression, lower survival probability
GTPBP1	1.61E-02	4.35E-02	LM	low expression, lower survival probability
GPR39	1.63E-02	4.35E-02	HM	high expression, lower survival probability
RBBP8	1.87E-02	4.82E-02	HM	high expression, lower survival probability
POSTN	1.97E-02	4.93E-02	HM	high expression, lower survival probability

Results of log rank regression analysis of survival estimates of below and above median expression of genes of interest. FDR = False Discovery Rate controlled p-value, LM = low-malignant, HM = high-malignant, Direction of expression and survival probability = indication whether low or high expression of the gene of interest results in a lower survival probability for grade III astrocytoma patients.

Supplementary Table 9: Linear regression results of final set of GFAP-regulated high- and low-malignant genes

See Supplementary File 2.
